# C1q^+^ Macrophage–Tumor Cell Interaction Promoted Tumorigenesis via GPR17/PI3K/AKT Pathway Induced DNA Hypermethylation in Nasopharyngeal Carcinoma

**DOI:** 10.1002/advs.202503434

**Published:** 2025-04-02

**Authors:** Yunzhi Liu, Cuicui Huang, Min Luo, Wenfu Lu, Baifeng Zhang, Lu Bai, Shuyue Zheng, Yanan Tan, Shanshan Li, Huali Wang, Lanqi Gong, Xinyuan Guan

**Affiliations:** ^1^ Clinical Oncology Center Shenzhen Key Laboratory for cancer metastasis and personalized therapy The University of Hong Kong‐Shenzhen Hospital Shenzhen 518053 China; ^2^ Advanced Energy Science and Technology Guangdong Laboratory Huizhou 516001 China; ^3^ Department of Clinical Oncology Li Ka Shing Faculty of Medicine The University of Hong Kong Hong Kong 999077 China; ^4^ Department of Surgery Division of Otolaryngology, Head and Neck Surgery The University of Hong Kong‐Shenzhen Hospital Shenzhen 518053 China; ^5^ MOE Key Laboratory of Tumor Molecular Biology Jinan University Guangzhou 510632 China

**Keywords:** C1q, DNA hypermethylation, nasopharyngeal carcinoma, PI3K/AKT signaling, tumor‐associated macrophage

## Abstract

Nasopharyngeal carcinoma (NPC) is one of the common head and neck cancers in Southern China and Southeast Asia. Although current studies have adequately characterized the tumor microenvironment (TME) of NPC, little attention has been paid to how cell‐cell interactions within the TME promote tumorigenesis. In this study, it is found that C1q^+^ tumor‐associated macrophages (TAMs) are significantly enriched in NPC tumors. Moreover, both enriched C1q^+^ TAMs and elevated C1q expression are associated with the progression and poor prognosis in NPC patients. In vitro and in vivo studies demonstrate that C1q directly boosts the malignancy and stemness of tumor cells. Mechanistically, C1q activates the Phosphatidylinositol‐3‐kinase (PI3K)/AKT pathway through interacting with GPR17, a member of the G protein‐coupled receptor family, thereby inducing DNA hypermethylation of tumor cells to promote tumor development. It is further proved that DNA hypermethylated NPC cells induced by C1q elicited the immunosuppressive phenotype of TAMs. Targeted blockade of C1q with a neutralizing antibody restricts NPC progression in the humanized mouse model. It is assumed that the differentiation of C1q^+^ TAMs possibly acquired both M1 and M2 polarization conditions. These findings provide new insights into the cellular communication in the TME of NPC and may have important applications for the development of new targeted therapies.

## Graphical abstract

In the TME of NPC, C1q secreted by C1q^+^ TAMs interacted with GPR17 to activate PI3K/AKT signaling through strengthening GPR17 coupling PI3K and increasing calcium levels in tumor cells. The activated PI3K/AKT signaling further induces DNA hypermethylation to promote the malignancy and stemness of tumor cells. Moreover, DNA hypermethylated NPC cells induced by C1q elicit the immunosuppressive phenotype of TAMs.

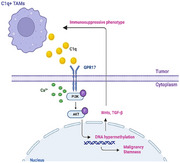

## 1 Introduction

Nasopharyngeal carcinoma (NPC) is a common subtype of head and neck cancer that is geographically distributed in Southern China and Southeast Asia.^[^ ^1^ ^]^ The initiation and development of NPC are closely related to Epstein–Barr virus‐mediated chronic inflammation, which results in abundant immune cell infiltration and shapes the complex tumor microenvironment (TME) of NPC.^[^ ^2^ ^]^ With the popularization and development of single‐cell RNA sequencing (scRNA‐seq) technology, various studies have jointly depicted the TME characteristics of NPC, whose stromal cell component mainly includes lymphocytes, myeloids, and fibroblasts.^[^ ^3^, ^4^ ^]^ Our previous study revealed the phenotypic abundance, immune dynamics, and developmental trajectory in the TME of NPC.^[^ ^3^ ^]^ Chen et al have also suggested potential biomarkers in the TME for anticancer treatment and risk stratification.^[^ ^4^ ^]^ Although current studies have adequately described the TME characteristics of NPC, little attention has been paid to how cell‐cell interactions within the TME promote NPC tumorigenesis and its underlying molecular mechanisms. We have identified that NPC tumor cells enforced the lipid‐driven development and homeostasis of Tregs,^[^ ^5^ ^]^ whereas the communication and interactions between tumor cells and other stromal cells, such as myeloid cells and fibroblasts, remain unknown. Therefore, in‐depth investigation of intercellular communication in the NPC TME will broaden our understanding of the pathogenesis and therapy of NPC.

Tumor‐associated macrophages (TAMs) are the most dominant member of the myeloid cells in the TME. Clinical correlative data and the plethora of preclinical studies in mouse models have shown that TAMs predominantly promote tumor progression.^[^ ^6^ ^]^ Notably, a novel subpopulation of TAMs, which was identified as C1q^+^ TAMs, has been discovered. C1q^+^ TAMs were found enriched in various cancers, and the infiltration of C1q^+^ TAMs in the TME was associated with tumor metastasis, recurrence, immunotherapy resistance, and was negatively correlated with patient survival,^[^ ^7^ ^a,b]^ indicating that C1q^+^ TAMs should play an important role in driving tumorigenesis. C1q^+^ TAMs have been shown to reinforce the expression of inhibitory receptors in cytotoxicity T cells and promote cytotoxicity T cell dysfunction.^[^ ^8^ ^]^ Nonetheless, the crosstalk between C1q^+^ TAMs and tumor cells has not been fully explored.

C1q is the recognition molecule of the complement classical pathway, which is predominantly secreted by the monocyte/macrophage system.^[^ ^9^ ^]^ In addition to acting as a complement molecule, C1q also interacts with various membrane signal receptors to activate multiple signaling pathways. C1q^+^ TAMs are the major source of C1q in the TME, and the expression of C1q was also correlated to the worse prognosis in tumor patients,^[^ ^7^, ^10^ ^]^ raising the question of whether C1q is just a biomarker of C1q^+^ TAMs or a driver for tumorigenesis. Indeed, C1q has been found to modulate the mitochondrial metabolism of CD8^+^ T cells and induce the CD8^+^ T cells differentiation into memory T cells rather than effector cells.^[^ ^11^ ^]^ Furthermore, C1q suppressed the activation of T cells in vitro.^[^ ^12^ ^]^ Research on the direct effect of C1q on tumor cells is controversial. C1q‐deficient mice have been reported to exhibit impaired tumor proliferation, adhesion, and migration, and this effect is not attributable to differences in tumor‐infiltrating immune cells.^[^ ^13^ ^]^ In addition, C1q facilitated the malignant phenotype of tumor cells in melanoma, pancreatic ductal adenocarcinoma, and hepatocellular carcinoma in vitro.^[^ ^7^, ^13^, ^14^ ^]^ However, C1q exhibited a cytotoxicity effect in an ovarian cancer cell line and a prostate cancer cell line.^[^ ^15^ ^]^ Despite the conflicting results, the role of C1q in NPC development is unknown. In this study, we discovered that C1q^+^ TAMs were enriched in NPC tumor TME. The relation between C1q expression and tumor progression was explored by bioinformatics analysis. In vitro and in vivo investigations further demonstrated that the crosstalk between tumor cells and C1q^+^ TAMs through secreted C1q. Our study identified C1q as a novel intermediary linked to the interaction between tumor cells and TAMs, which might be a new therapeutic target for NPC treatment.

## 2 Results

### 2.1 TAMs Secreted C1q is Associated with NPC Development

**Figure 1:**
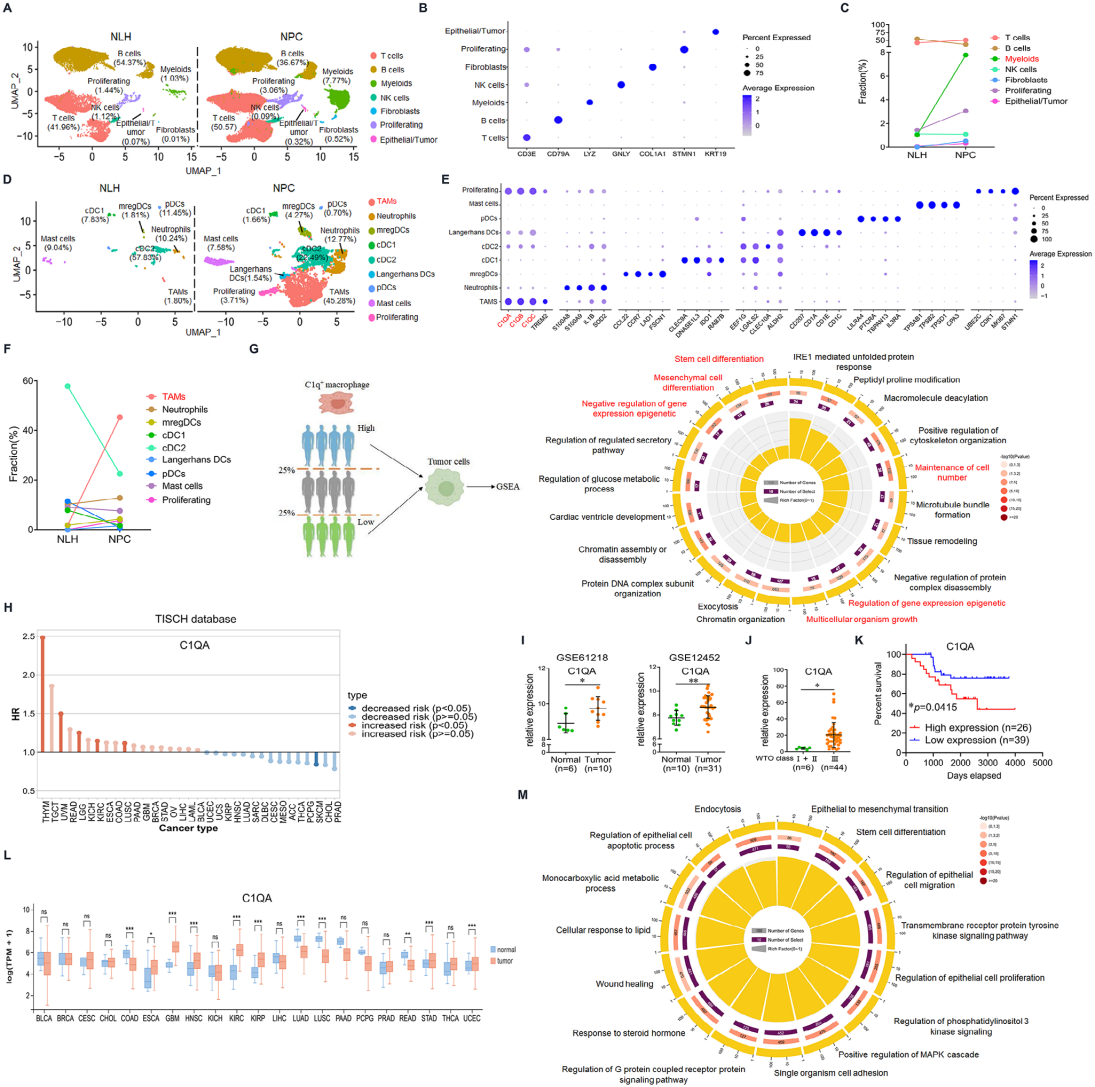
TAMs secreted C1q was associated with NPC development. A‐G) Tissues from NPC and NLH patients were conducted for scRNA‐seq. (A) UMAP displayed the major cell components. (B) Mark genes of each cluster were shown. (C) The fraction of each cell lineage has been calculated. (D) UMAP displayed the subclusters in the Myeloids. (E) Mark genes of each subcluster in the Myeloids were shown. (F) The fraction of each subcluster in the Myeloids has been exhibited. (G) GO pathway enrichment was performed to identify differential pathways between C1q^+^ TAMs highly enriched NPC patients and patients with low C1q^+^ TAMs enrichment. C1q^+^ TAMs high and low were divided at the upper quartile and lower quartile. H) Survival analysis of pan‐cancer scRNA‐seq data with high C1QA expression and low C1QA expression based on the TISCH database. I) Expression levels of C1QA in tissues from NPC patients and non‐tumor individuals in the GSE61218 and GSE12452 datasets. J) Expression levels of C1QA in tissues from NPC tissues classified as stage III and tissues from patients classified as stage I and II in the EGAD00001009047 dataset. K) Survival analysis of NPC patients with high C1QA expression and low C1QA expression. L) Expression levels of C1QA in pan‐cancer analysis based on the TCGA database. M) GO pathway enrichment was performed to identify common differential pathways between C1q high expression patients and C1q low expression patients in TCGA datasets. Data on breast cancer, cholangiocarcinoma, and hepatocellular carcinoma were included. C1q high expression and low expression were divided at the upper quartile and the lower quartile. ^*^ *p* < 0.05, ^**^ *p* < 0.01. Data from one representative experiment of three independent experiments are presented. Kaplan–Meier and log‐rank tests were used to determine survival rates.

To explore the TME character of NPC, 10×scRNA‐seq analysis was performed to identify the difference in RNA expression profiles between NPC tumor tissues and nasopharyngeal lymphatic hyperplasia (NLH) counterparts at single‐cell resolution. Seven major cell lineages were found in NPC tissues (**Figure** **1**A,B). Among them, myeloid cells were significantly enriched in NPC tissues compared to NLH tissue (Figure 1C). We then identified subpopulations of myeloid cells and found that TAMs were mostly abundant, which were characterized by secreting the complement molecule C1q (Figure 1D–F). The majority of C1q in the TME was predominantly secreted by TAMs (Figure S2A, Supporting Information), with nearly all TAMs demonstrating C1q‐producing capacity (Figure S2B, Supporting Information), indicating that TAMs serve as the primary source of C1q in the TME. To further predict the relation between C1q^+^ TAMs and tumorigenesis, GO pathway enrichment was performed between tumor cells from C1q^+^ TAMs highly enriched patients and C1q^+^ TAMs low enriched patients. Pathways of “Multicellular organism growth”, “Mesenchymal cell differentiation”, “Stem cell differentiation”, and “Gene expression epigenetic” were discovered, which indicated C1q^+^ TAMs might regulate malignancy, stemness, and epigenetic change of tumor cells (Figure 1G). Pan‐cancer single‐cell data were analyzed in the TISCH database. C1q expression was found to accumulate in macrophages in almost all the cancers (Figure S1, Supporting Information), and high C1q expression was associated with poor prognosis in five types of cancers (Figure 1H; Figure S2C, Supporting Information).

Since C1q was the most significant secreted protein of TAMs and was predominantly secreted by macrophages, we hypothesized that C1q acted as the intermediary linking the crosstalk between TAMs and tumor cells. Therefore, we analyzed the relationship between C1q expression and tumor development. Elevated C1q expression was found in NPC tumor tissues compared to normal counterparts (Figure 1I; Figure S2D, Supporting Information). Similarly, tumor tissues obtained from patients classified as stage III exhibited higher C1q levels than those from NPC patients classified as stage I and II (Figure 1J; Figure S2E, Supporting Information). Furthermore, Cox regression analysis identified C1q expression as an independent prognostic factor for NPC patients, and high C1q levels led to a poor prognosis (Figure 1K; Figure S2F, Supporting Information). We also discovered the C1q expression pattern in pan‐cancer in the TCGA database (Figure 1L; Figure S2G, Supporting Information) and identified common differential pathways between C1q high expression patients and C1q low expression patients. These pathways involved cell proliferation, epithelial‐mesenchymal transition (EMT), stem cell differentiation, and molecular signaling pathways containing PI3K, MAPK, and G protein‐coupled receptor (GPCR) pathways (Figure 1M). Above all, these data indicated that TAMs secreted C1q was associated with NPC development and C1q might regulate the malignancy, stemness, and epigenetic change of tumor cells.

### 2.2 C1q Enhances the Malignant and Stemness Phenotype of NPC Cells

**Figure 2:**
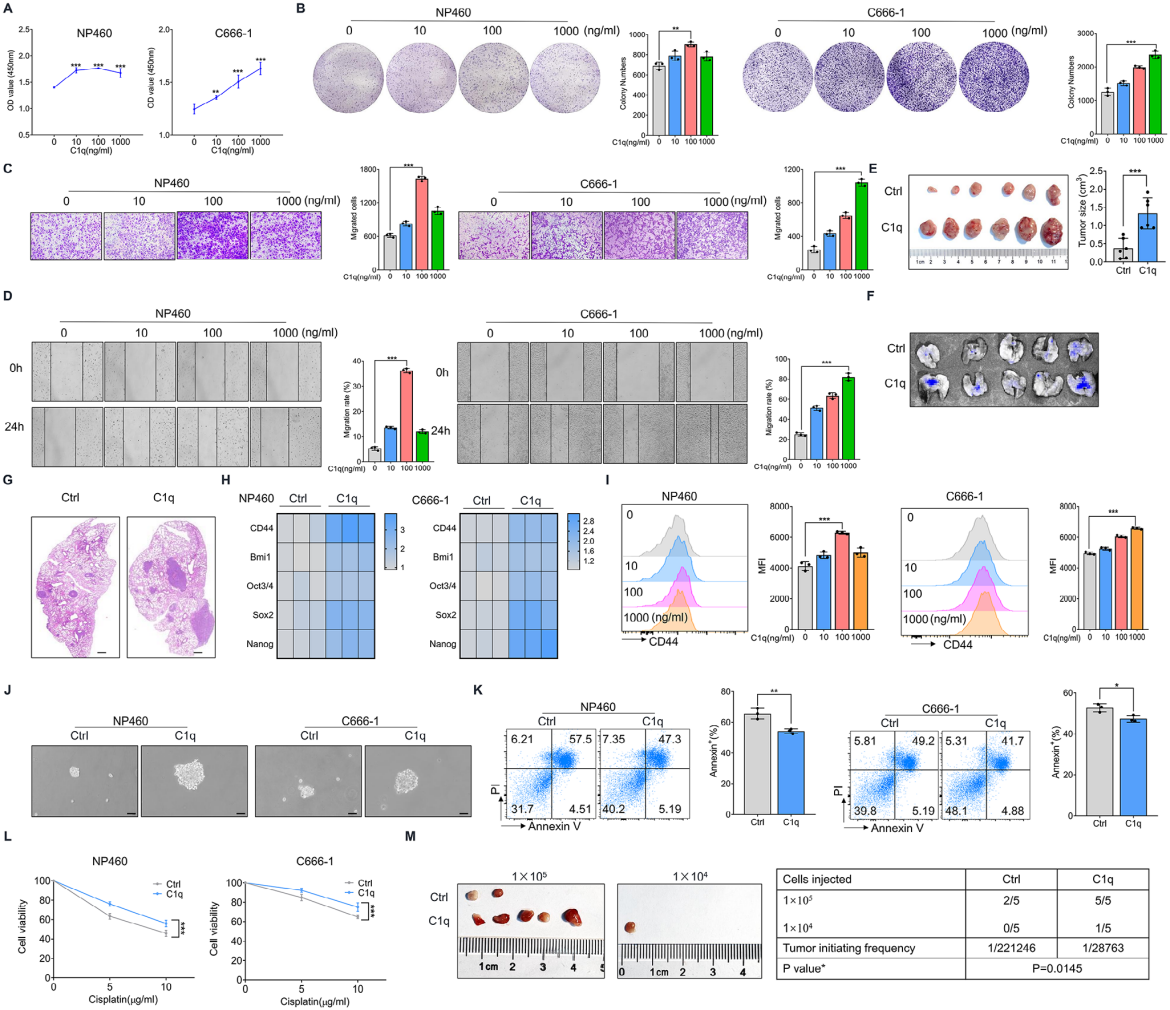
C1q enhanced the malignant and stemness phenotype of NPC cells. A) NP460 and C666‐1 cells were treated with indicated concentrations of C1q for 48 h, the CCK‐8 assay was performed to detect cell proliferation. B) NP460 and C666‐1 cells were treated with indicated concentrations of C1q for 7 days, the colony formation assay was used to determine cell proliferation. C,D) NP460 and C666‐1 cells were treated with indicated concentrations of C1q for 24 h. The migration test (C) and wound healing assays (D) were performed to detect the migration ability. E) C666‐1 cells were treated with 1 µg mL^−1^ C1q or equal volume PBS as counterparts for three days in vitro and then subcutaneously injected into nude mice for three weeks. Tumor size was measured (n = 6). F,G) GFP‐labeled C666‐1 cells were treated with 1 µg mL^−1^ C1q and tail vein injected into nude mice for four weeks (n = 5). (F) GFP fluorescence was detected in lung tissues by live animal imaging to indicate tumor nodules. (G) H&E staining was performed to evaluate the pathological changes in lung tissues. Scale bars = 500 µm. H,I) NP460 and C666‐1 cells were treated with 1 µg mL^−1^ C1q for 24 h. (H) RT‐qPCR analysis was conducted to detect the mRNA levels of CSC‐related genes. (I) Flow cytometry analysis was performed to detect CD44 expression. J) NP460 and C666‐1 cells were cultured under 100 × N2, 50 × B27, 5 µg mL^−1^ insulin, 20 ng mL^−1^ EGF, 10 nM FGF and 0.4% BSA conditions for 7 days, scale bars = 50 µm. K) NP460 and C666‐1 cells were treated with 10 µg mL^−1^ cisplatin and 1 µg mL^−1^ C1q for 24 h. The flow cytometry assay was used to evaluate cell apoptosis. L) NP460 and C666‐1 cells were treated with indicated concentrations of cisplatin and 1µg mL^−1^ C1q for 24 h. The CCK‐8 assay was conducted to measure cell viability. M) C666‐1 cells were treated with 1 µg mL^−1^ C1q for three days in vitro and subcutaneously injected into nude mice for three weeks; the tumor initiation frequency of CSCs was calculated (n = 5). ^*^ *p* < 0.05, ^**^ *p* < 0.01, ^***^ *p* < 0.001. Data from one representative experiment of three independent experiments are presented. Two‐tailed unpaired Student's *t*‐test was used to analyze the difference between the two groups. One‐way ANOVA was used to compare the differences among multiple groups.

We first evaluated the direct effect of C1q on NPC cell malignancy. Nasopharyngeal epithelial cells (NP460) and nasopharyngeal tumor cells (C666‐1) were treated with C1q protein purified from pooled normal human plasma by the CompTech company. CCK‐8 and colony formation assays indicated that C1q boosted the proliferation of both NP460 and C666‐1 cells (**Figure** **2**A,B). Intriguingly, the Edu assay showed that C1q promoted DNA synthesis not only in the complete culture medium (Figure S3A, Supporting Information), but also in FBS‐free conditions (Figure S3B, Supporting Information). Migration assays were performed to clarify the role of C1q in cell migration, and the results showed that C1q treatment facilitated more cells migrating to the lower chamber compared to the controls (Figure 2C). Similarly, increased wound‐healing efficacy was observed in cells treated with C1q (Figure 2D). Moreover, C1q treatment promoted the expression of EMT markers (Figure S3C, Supporting Information). Next, we treated NPC tumor cells with C1q for three days in vitro and then subcutaneously or tail‐vein injected them into nude mice. The xenograft model demonstrated that C1q treatment boosted tumorigenesis in subcutaneous tumors compared to the controls (Figure 2E). Furthermore, the lung metastasis model also suggested that more severe tumor nodules were enriched in the lung when tumor cells were exposed to C1q (Figure 2F,G).

Cancer stem cells (CSCs) are unique seeds capable of tumor initiation, enabling tumor cells to self‐renew and mediating anticancer drug resistance. We then investigate whether C1q regulates the stemness of NPC cells. RT‐qPCR assays demonstrated that stemness‐related gene levels were increased in C1q‐treated cells compared to controls (Figure 2H). As CD44 is a widely regarded CSC marker, CD44 expression was estimated by flow cytometry, and the results showed that C1q treatment increased CD44 expression in NPC cells (Figure 2I). The self‐renewal ability of NPC cells was evaluated by the sphere formation assay, and increased sphere diameters were exhibited in cells treated with C1q (Figure 2J). Resistance to anticancer drugs is another feature of CSCs. Cisplatin, one of the most common chemotherapeutic agents, was chosen to induce NPC cell apoptosis. Annexin V‐PI staining and CCK‐8 assays demonstrated that C1q treatment reinforced the drug‐resistance ability of NPC cells (Figure 2K,L). A limited dilution assay was used to estimate tumor stemness in vivo. The results showed that as few as 28763 C1q‐treated tumor cells were sufficient to form tumors in nude mice, whereas eight times more control cells were required to generate tumors (Figure 2M). Taken together, these results indicated that C1q enhanced the malignant and stemness phenotype of NPC cells.

### 2.3 C1q Elicits DNA Hypermethylation of NPC Cells

**Figure 3:**
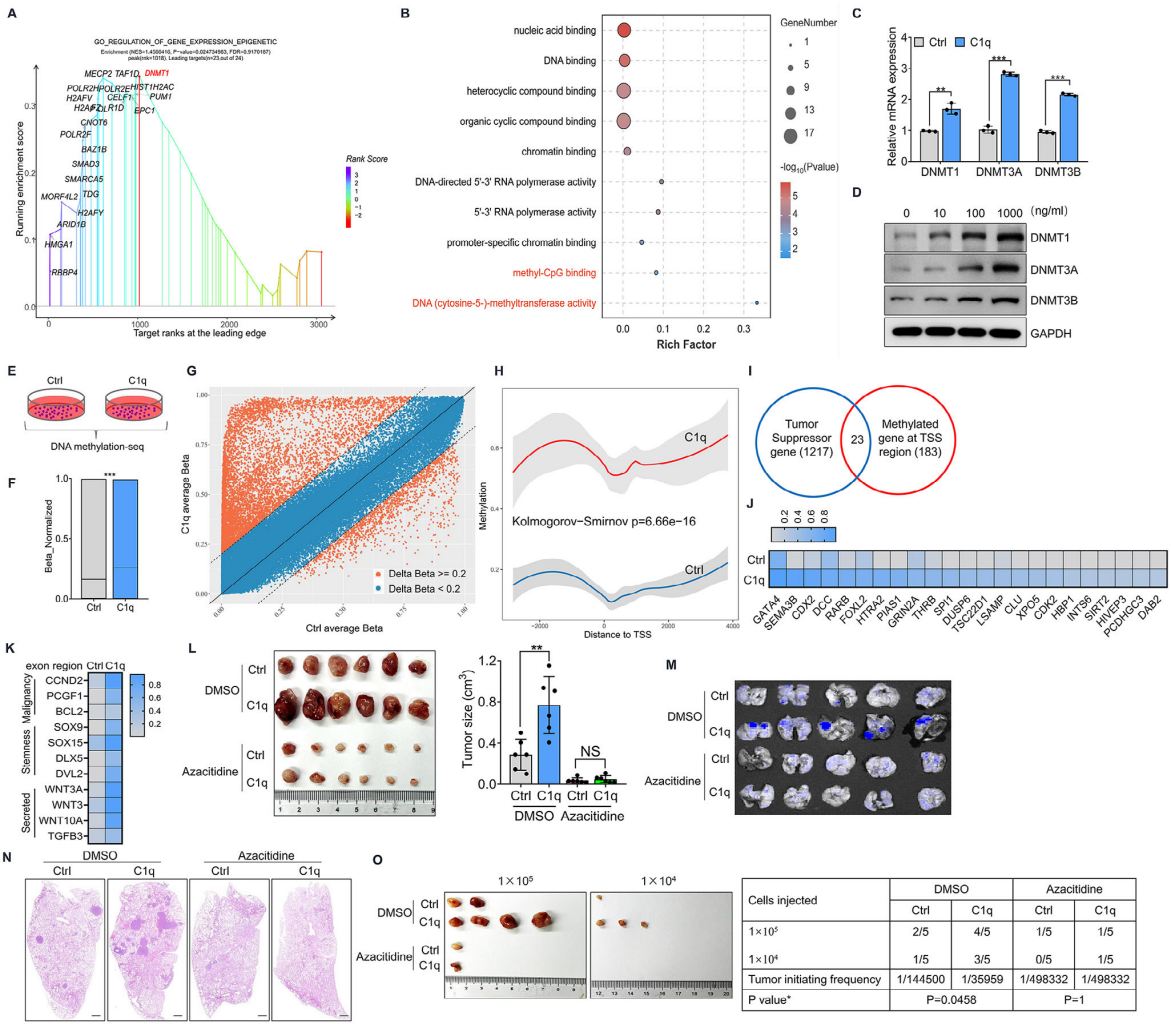
C1q elicited DNA hypermethylation of NPC cells. A) Differential genes in the “Regulation of gene expression epigenetic” pathway were displayed based on our scRNA‐seq data. B) Pathway enrichment of differential genes was performed. C) C666‐1 cells were treated with 1 µg mL^−1^ C1q for 24 h, RT‐qPCR analysis was performed to detect DNMTs expression. D) C666‐1 cells were treated with indicated concentrations of C1q for 24 h, the expression of DNMTs was determined by western blot analysis. E–K) C666‐1 cells were treated with 1 µg mL^−1^ C1q for 24 h to conduct DNA methylation‐seq. E) Diagram of cell treatment. F) Normalized β‐value was displayed. G) The average β‐value of each DNA site was shown. H) The distance of the DNA methylation site to the TSS region was exhibited. I) Venn diagram of tumor suppressor genes in TSGene database and differentially methylated genes at TSS region based on DNA methylation‐seq. J) Heatmap displayed the common genes between tumor suppressor genes in the TSGene database and differentially methylated genes at the TSS region based on DNA methylation‐seq. K) Heatmap displayed the differential genes methylated at the exon region. L) C666‐1 cells were treated with 1 µg mL^−1^ C1q for three days in vitro and then subcutaneously injected into nude mice for four weeks. Tumor size was measured. In some cases, cells were cultured in the condition of 5 µM azacitidine (n = 6). M,N) GFP‐labeled C666‐1 cells were treated with 1 µg mL^−1^ C1q and tail vein injected into nude mice for four weeks. In some cases, cells were cultured in the condition of 5 µM azacitidine (n = 5). (M) GFP fluorescence was detected in lung tissues by live animal imaging to indicate tumor nodules. (N) H&E staining was performed to evaluate the pathological changes in lung tissues. Scale bars = 500 µm. O) C666‐1 cells were treated with 1 µg mL^−1^ C1q for three days in vitro and subcutaneously injected into nude mice for 4 weeks; the tumor initiation frequency of CSCs was calculated. In some cases, cells were cultured in the condition of 5 µM azacitidine (n = 5). NS: not significant, ^**^ *p* < 0.01, ^***^ *p* < 0.001. Data from one representative experiment of three independent experiments are presented. Two‐tailed unpaired Student's *t*‐test was used to analyze the difference between the two groups.

The pathway enrichment of our single‐cell data suggested that C1q may mediate the epigenetic regulation of NPC tumor cells. In vivo nude mice experiment also showed that tumor cells treated with C1q in vitro exhibited promoted tumor progression in vivo. Thus, we then investigated how C1q regulated tumor development through epigenetic regulation. GSEA analysis indicated that DNMT1, a DNA methyltransferase, exhibited the highest enrichment score (**Figure** **3**A), and differential genes were also enriched in the DNA methylation pathway (Figure 3B), suggesting that C1q may regulate tumor cell DNA methylation through DNA methyltransferases (DNMTs). We proved that C1q promoted both canonical maintenance enzyme DNMT1 and de novo DNMTs 3A and 3B expressions at both transcriptional and translational levels (Figure 3C,D). To further investigate how C1q elicited DNA hypermethylation regulating tumor development, we performed DNA methylation‐seq on C1q‐treated tumor cells (Figure 3E). The results showed higher β‐value levels in tumor cells treated with C1q (Figure 3F) and more DNA hypermethylation sites (Figure 3G) compared to controls. DNA methylation around the transcription start site (TSS) region blocked transcription, while methylation around the exon region reinforced transcription. Therefore, we analyzed the genome location of the differential methylation site between C1q‐treated cells and control cells (Figure S4A,B, Supporting Information). We found aggravated DNA methylation levels around the TSS region in C1q‐treated tumor cells (Figure 3H), and almost 1/8 methylation sites belonged to widely known tumor suppressor genes (Figure 3I,J). Moreover, various malignancy and stemness‐related genes were found methylated around the exon region. Notably, genes of the WNTs family and transforming growth factor‐beta (TGF‐β) were also shown to be methylated around the exon region, indicating that DNA hypermethylated tumor cells, induced by C1q, may regulate the tumor microenvironment through boosted expression of secreted proteins (Figure 3K; Figure S4C, Supporting Information). We further confirmed that C1q limited the expression of various tumor suppressor genes, while promoted the expression of oncogenes and secreted cytokines (Figure S4D, Supporting Information). Our findings suggest that C1q played a crucial role in regulating tumor development through epigenetic regulation of DNA methylation.

To further demonstrate that C1q promoted tumor progression by eliciting DNA hypermethylation of tumor cells, NPC tumor cells were cultured with C1q in the presence or absence of Azacitidine, a DNA methylation inhibitor, for three days in vitro. The treated tumor cells were then injected into nude mice for the xenograft model, the lung metastasis model, and the limited dilution assay. The results showed that Azacitidine treatment abolished the effect of C1q on tumor development in the xenograft model (Figure 3L). Additionally, no significant differences in metastatic tumor nodules were detected between C1q‐treated cells and control cells in the presence of Azacitidine (Figure 3M,N). Furthermore, comparable amounts of tumor cells were required to generate tumors when C1q‐treated cells and controls were cultured in a medium containing Azacitidine (Figure 3O). These findings confirm that C1q promoted tumor progression by eliciting DNA hypermethylation of NPC cells.

### 2.4 C1q Promotes Tumorigenesis of NPC Cells Through PI3K‐AKT Signaling

**Figure 4:**
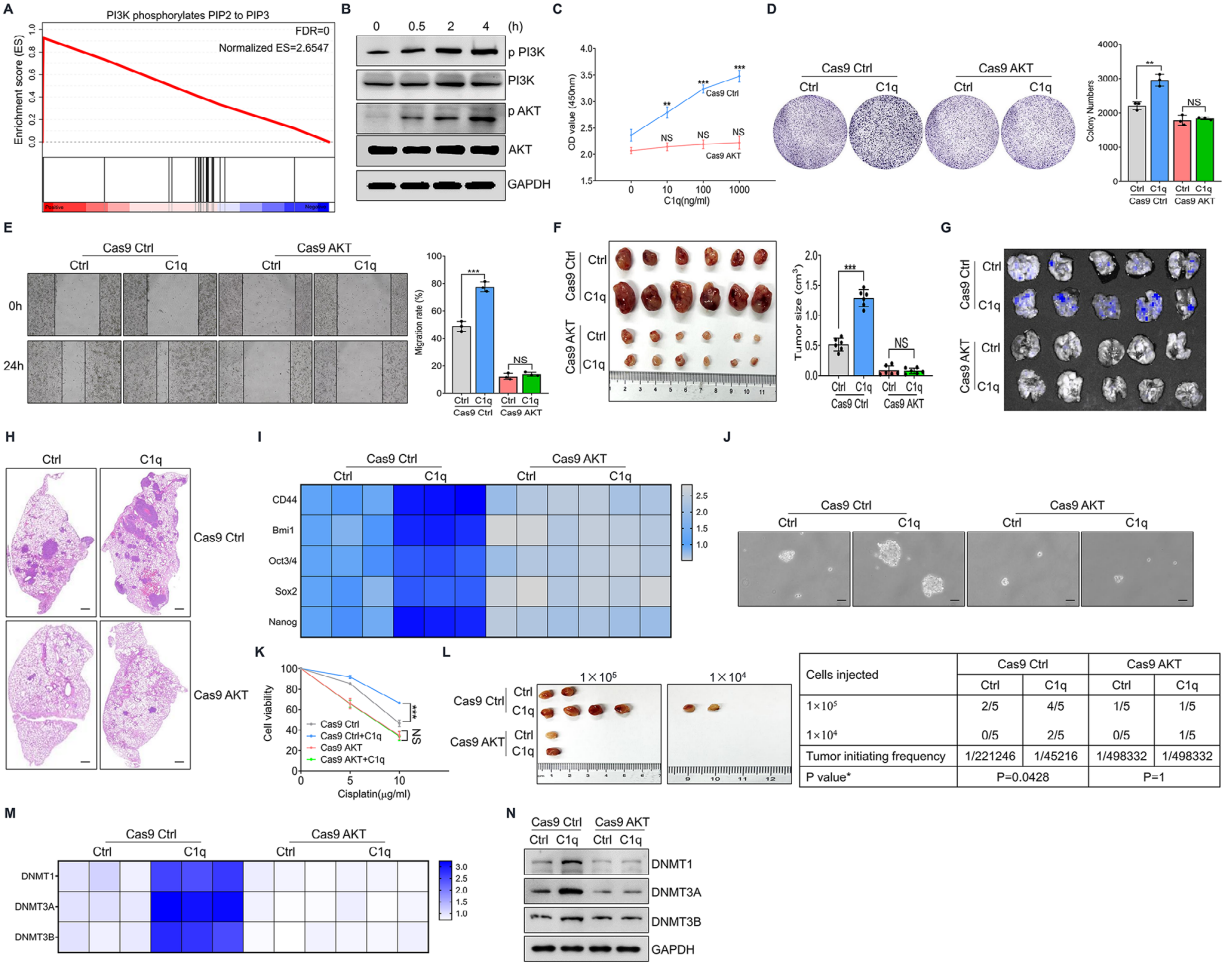
C1q promoted the tumorigenesis of NPC cells through PI3K‐AKT signaling. A) C666‐1 cells were treated with 1 µg mL^−1^ C1q for 24 h, then were processed with bulk RNA‐seq. The most significant differential pathway was enriched by the GSEA analysis in the Reactome dataset. B) C666‐1 cells were treated with 1 µg mL^−1^ C1q. Phosphorylation of PI3K and AKT was determined by western blot analysis. C) C666‐1 cells were treated with C1q for 48 h, the CCK‐8 assay was performed to detect cell proliferation. D) Indicated cells were treated with 1 µg mL^−1^ C1q for 7 days. The colony formation assay was used to determine cell proliferation. E) The wound healing assay was performed to detect the migration ability. F) C666‐1 cells were treated with 1 µg mL^−1^ C1q for three days in vitro and then subcutaneously injected into nude mice for four weeks. Tumor size was measured (n = 6). G,H) GFP‐labeled indicated cells were treated with 1 µg mL^−1^ C1q and tail vein injected into nude mice for four weeks (n = 5). (G) GFP fluorescence was detected in lung tissues by animal live imaging to indicate tumor nodules. (H) H&E staining was performed to evaluate the pathological changes in lung tissues. Scale bars = 500 µm. I) Indicated C666‐1 cells were treated with 1 µg mL^−1^ C1q for 24 h. RT‐qPCR analysis was conducted to detect the mRNA levels of CSC‐related genes. J) Indicated C666‐1 cells were cultured under 100 × N2, 50 × B27, 5 µg mL^−1^ insulin, 20 ng mL^−1^ EGF, 10 nM FGF, and 0.4% BSA conditions for 7 days, scale bars = 50 µm. K) Indicated C666‐1 cells were treated with cisplatin for 24 h. The CCK‐8 assay was conducted to measure cell viability. L) Indicated cells were treated with 1 µg mL^−1^ C1q for three days in vitro and subcutaneously injected into nude mice for 4 weeks; the tumor initiation frequency of CSCs was calculated (n = 5). M,N) Indicated were treated with 1 µg mL^−1^ C1q for 24 h. (M) RT‐qPCR analysis was performed to detect DNMTs expression. (N) The expression of DNMTs was determined by western blot analysis. NS: not significant, ^**^ *p* < 0.01, ^***^ *p* < 0.001. Data from one representative experiment of three independent experiments are presented. Two‐tailed unpaired Student's *t*‐test was used to analyze the difference between the two groups. One‐way ANOVA was used to compare the differences among multiple groups.

To investigate the molecular mechanism of C1q in NPC cell regulation, C1q‐treated cells and control cells were subjected to bulk RNA‐Seq. Reactome pathway enrichment was performed to identify differential pathways, and the PI3K pathway was found to be the most significant differential pathway (**Figure** **4**A), indicating that C1q might activate the PI3K‐AKT pathway. We validated C1q‐induced PI3K and AKT phosphorylation in vitro (Figure 4B). To further demonstrate whether C1q regulated NPC cells through PI3K‐AKT signaling, AKT‐deficient tumor cells were established using the CRISPR‐Cas9 system (Figure S5A, Supporting Information). We found that AKT deficiency abolished the promotive effect of C1q on NPC cell proliferation (Figure 4C,D). Similarly, C1q exhibited limited influence on tumor cell migration in AKT knockout conditions (Figure 4E). Moreover, AKT ablation dismissed the effect of C1q on tumorigenesis and distant metastasis in the nude mice xenograft model and the lung metastasis model (Figure 4F–H). We then investigated whether C1q regulated the stemness of NPC cells through AKT signaling. Comparable stemness‐related gene expressions were detected in C1q‐treated cells and controls when AKT expression was knocked out (Figure 4I). Consistently, the effect of C1q on sphere formation was abolished in AKT‐knockout NPC cells (Figure 4J). As indicated by the CCK8 assay, AKT knockout abolished the regulatory effect of C1q on NPC cells resistant to cisplatin treatment (Figure 4K). The in vivo limited dilution assay also exhibited an impaired effect of C1q on tumor development when AKT expression was terminated (Figure 4L). Moreover, we found that AKT ablation dismissed the role of C1q in promoting tumor cell DNA methylation (Figure 4M,N). Overall, these data suggested that C1q promoted the tumorigenesis of NPC cells through PI3K‐AKT signaling.

### 2.5 C1q Activates PI3K‐AKT Signaling Through Interaction with Membrane GPR17

**Figure 5:**
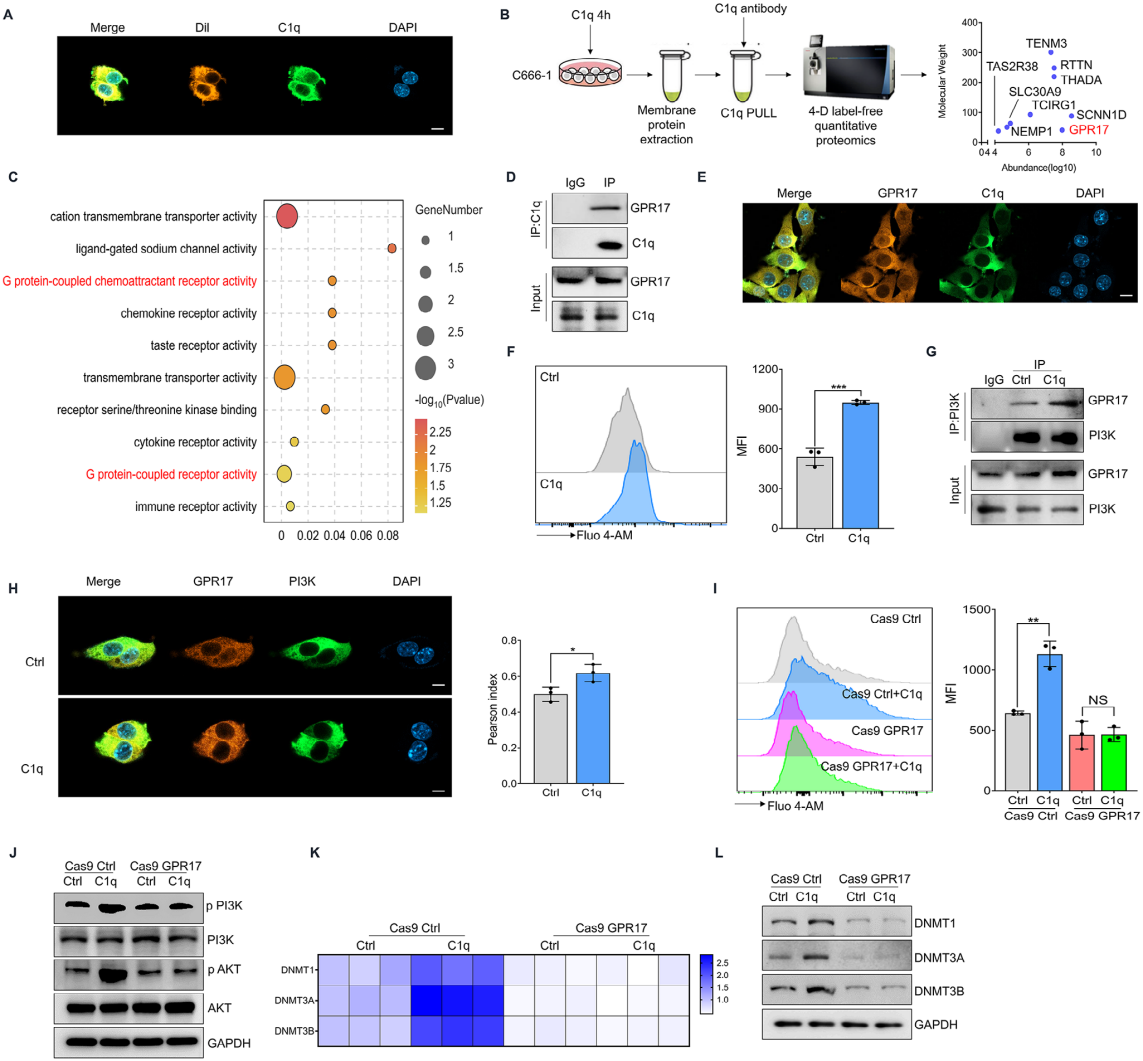
C1q activated PI3K‐AKT signaling through interaction with membrane GPR17. A) C666‐1 cells were treated with 1 µg mL^−1^ C1q for 4 h. The immunofluorescence assay was performed to assess the localization of C1q. Dil was stained to identify the cell membrane. B) C666‐1 cells were treated with 1 µg mL^−1^ C1q for 4 h. Membrane protein was extracted, and the C1q‐interacting protein was pulled down by anti‐C1q antibody. The precipitants were performed with 4‐D label‐free quantitative proteomics to identify C1q coupled protein. C) Pathway enrichment was conducted in the C1q‐interacting proteins. D–H) C666‐1 cells were treated with 1 µg mL^−1^ C1q for 4 h. (D) The immunoprecipitation assay was performed to analyze the interaction between C1q and GPR17. (E) The interaction between C1q and GPR17 was detected by the immunofluorescence assay. (F) Cellular calcium level was evaluated using flow cytometry analysis. (G) The interaction between GPR17 and PI3K was detected by the immunoprecipitation assay. (H) The immunofluorescence assay was performed to assay the interaction between GPR17 and PI3K. I,J) Indicated cells were treated with 1 µg mL^−1^ C1q for 4 h. In some cases, the expression of GPR17 in C666‐1 cells was knocked down. (I) The flow cytometry analysis was performed to explore cellular calcium levels. (J) Activation of the PI3K/AKT pathway was detected by western blot analysis. K,L) Indicated cells were treated with 1 µg mL^−1^ C1q for 24 h. In some cases, the expression of GPR17 in C666‐1 cells was knocked down. (K) The expression of DNMTs was evaluated by RT‐qPCR analysis. (L) The expression of DNMTs was determined by western blot analysis. NS: not significant, ^*^ *p* < 0.05, ^**^ *p* < 0.01, ^***^ *p* < 0.001. Data from one representative experiment of three independent experiments are presented. Two‐tailed unpaired Student's *t*‐test was used to analyze the difference between the two groups.

C1q has been found to interact with various cell membrane proteins to activate signaling pathways. To investigate how C1q activates the PI3K‐AKT signaling, we examined the cellular location of C1q using Dil, a cell membrane dye. The immunofluorescence assay exhibited that C1q localized at the cell membrane (**Figure** **5**A), indicating that C1q may interact with a membrane receptor to activate the PI3K‐AKT signaling. To identify the receptor C1q interacted with, tumor cells were treated with C1q for 4 h in vitro, and then membrane proteins were extracted. The C1q immunoprecipitation complex was pulled down from the extracted membrane protein, and the immunoprecipitants were identified through quantitative proteomics. Among all the recognized proteins, GPR17, a member of the G protein‐coupled receptor (GPCR) family, was the only signal receptor (Figure 5B,C). The interaction between C1q and GPR17 was confirmed by immunoprecipitation (Figure 5D) and immunofluorescence assays (Figure 5E). GPR17 belongs to the G(i/o) subclass of the GPCRs family, which was found to activate PI3K through both increased intracellular messenger calcium levels and direct interaction with PI3K. We tested intracellular calcium levels using a calcium fluorescence probe and found that C1q‐treated tumor cells exhibited increased calcium levels compared to controls (Figure 5F). Moreover, C1q promoted the interaction between GPR17 and PI3K (Figure 5G,H). To further demonstrate that C1q‐induced PI3K‐AKT signaling activation through GPR17, GPR17 expression was ablated in tumor cells (Figure S5B, Supporting Information). Comparable calcium levels were found in C1q‐treated cells and controls when GPR17 expression was knocked down (Figure 5I). Furthermore, GPR17 ablation abolished the effect of C1q on PI3K‐AKT signaling activation (Figure 5J). The promotive effect of C1q on tumor cell DNA methylation was also restricted in the GPR17 knockdown condition (Figure 5K,L). Besides, there was no significant difference in GPR17 expression between NPC tumor tissues and control samples in clinical samples, indicating that the effect of GPR17 on NPC development depended on receptor activation (Figure S5C, Supporting Information). cC1QR, gC1QR and HMGB1 were the previously documented receptors of C1q. We discovered that both GPR17 and these receptors were expressed in the NPC RNA‐seq data (Figure S5D, Supporting Information), while only GPR17 knockout exhibited the most pronounced reduction in DNMTs expression (Figure S5E, Supporting Information), suggesting that GPR17 serves as a predominant regulator of DNA methylation mediated by C1q. In summary, our data suggested that C1q activated PI3K‐AKT signaling through interaction with the membrane receptor GPR17.

### 2.6 DNA Hypermethylated NPC Cells Induced by C1q Elicited the Immunosuppressive Phenotype of TAMs

**Figure 6:**
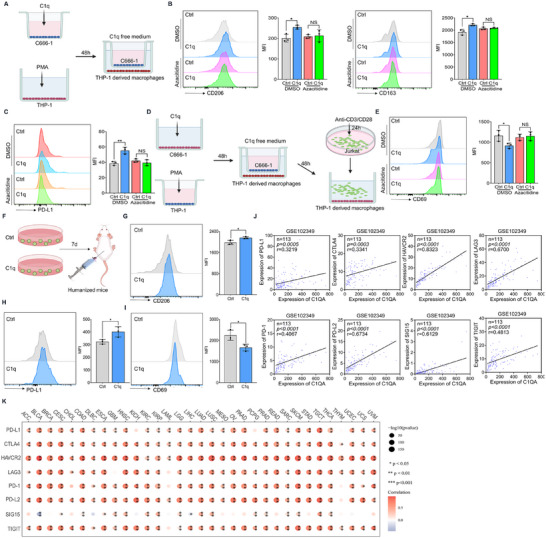
DNA hypermethylated NPC cells induced by C1q elicited an immunosuppressive phenotype of TAMs. A) THP‐1 cells were treated with 100 ng mL^−1^ PMA for 24 h to polarize into macrophages. Tumor cells were treated with 1 µg mL^−1^ C1q for 48 h in a 0.4 µm pore insert and then replaced with a refreshed C1q‐free medium. The inserts were cocultured with PMA‐derived macrophages for 48 h. In some cases, tumor cells were cultured in the condition of 5 µM azacitidine. B) The expression of CD206 and CD163 was evaluated by the flow cytometry analysis. C) The PD‐L1 level was determined by flow cytometry analysis. D) Jurkat cells were activated by 1 µg mL^−1^ anti‐CD3/CD28 antibody for 24 h, then cocultured with tumor cell cocultured macrophages for another 48 h. In some cases, tumor cells were cultured in the condition of 5 µM azacitidine. E) The expression level of CD69 was explored by the flow cytometry assay. F) C666‐1 cells were treated with 1 µg mL^−1^ C1q for seven days. The cells were then harvested and subcutaneously injected into humanized mice for three weeks (n = 3). G,H) The CD206 and PD‐L1 expression were determined by flow cytometry. The results were gated in CD68^+^ cells. I) Flow cytometry analysis was performed to detect CD69 levels in T cells. The results were gated in CD3^+^ cells. J) The correlation between C1QA expression and immune checkpoint expression in NPC patients was analyzed based on the GSE102349 dataset. K) The correlation between C1QA expression and immune checkpoint expression in pan‐cancer was analyzed based on the TCGA database. NS: not significant, ^*^ *p* < 0.05, ^**^ *p* < 0.01. Data from one representative experiment of three independent experiments are presented. Two‐tailed unpaired Student's *t*‐test was used to analyze the difference between the two groups.

We previously discovered that C1q‐induced genes of the WNTs family and TGF‐β methylation around the exon region in NPC cells. Since WNTs and TGF‐β both elicit the immunosuppressive phenotype of macrophages,^[^ ^16^ ^]^ we investigated whether DNA hypermethylated NPC cells induced by C1q would boost the immunosuppressive phenotype of macrophages in turn. NPC tumor cells were treated with C1q for 48 h in the cell insert, then cocultured with THP‐1‐derived macrophages in a C1q‐free condition (**Figure** **6**A). We observed elevated CD206 and CD163 levels in macrophages cocultured with C1q‐treated tumor cells compared to controls, while Azacitidine treatment abolished the effect of tumor cells to promote macrophage M2 phenotype transition (Figure 6B). Furthermore, macrophages cocultured with C1q‐treated tumor cells exhibited higher PD‐L1 expression than control cells in a DNA methylation‐dependent manner (Figure 6C). The cocultured macrophages were then incubated with activated T cells (Figure 6D). Impaired T cell activation was detected when T cells were incubated with macrophages that cocultured with DNA hypermethylated NPC cells induced by C1q, as indicated by a decline in CD69 expression, while comparable CD69 levels in T cells were observed when tumor cells were treated with Azacitidine (Figure 6E). Moreover, C1q has been reported to limit T cell activation directly. Therefore, we incubated activated T cells with C1q to confirm the direct effect of C1q on T cell activation (Figure S6A, Supporting Information). Indeed, C1q treatment restricted CD69 expression in T cells (Figure S6B, Supporting Information). To further demonstrate C1q‐treated NPC cells elicited a more immunosuppressive phenotype of TME in vivo. C1q‐treated NPC cells were subcutaneously injected into humanized mice (Figure 6F). We observed aggravated CD206 and PD‐L1 expressions in TAMs from C1q‐treated tumors compared to the counterparts (Figure 6G,H). Besides, a declined in CD69 level in T cells was also detected in C1q‐treated tumors (Figure 6I), suggesting an immunosuppressive phenotype of TME. We analyzed the correlation between C1q expression and various inhibitory immune checkpoints in NPC tissues. The results showed that C1q expression was positively correlated with the expression of inhibitory immune checkpoints such as PD‐L1, CTLA‐4, and PD‐1 (Figure 6J; Figure S6C, Supporting Information). Pan‐cancer analysis also showed that C1q expression is positively correlated with the expression of inhibitory immune checkpoints in almost all cancers, indicating that C1q acts as a profound inducer of an immunosuppressive tumor microenvironment (Figure 6K; Figure S6D, Supporting Information). Overall, our findings demonstrated that DNA hypermethylated NPC cells induced by C1q elicited an immunosuppressive phenotype of tumor‐associated macrophages, and C1q induced an immunosuppressive tumor microenvironment during tumor development.

### 2.7 Anti‐C1q Treatment Restricted NPC Development in the Humanized Mouse Model

**Figure 7:**
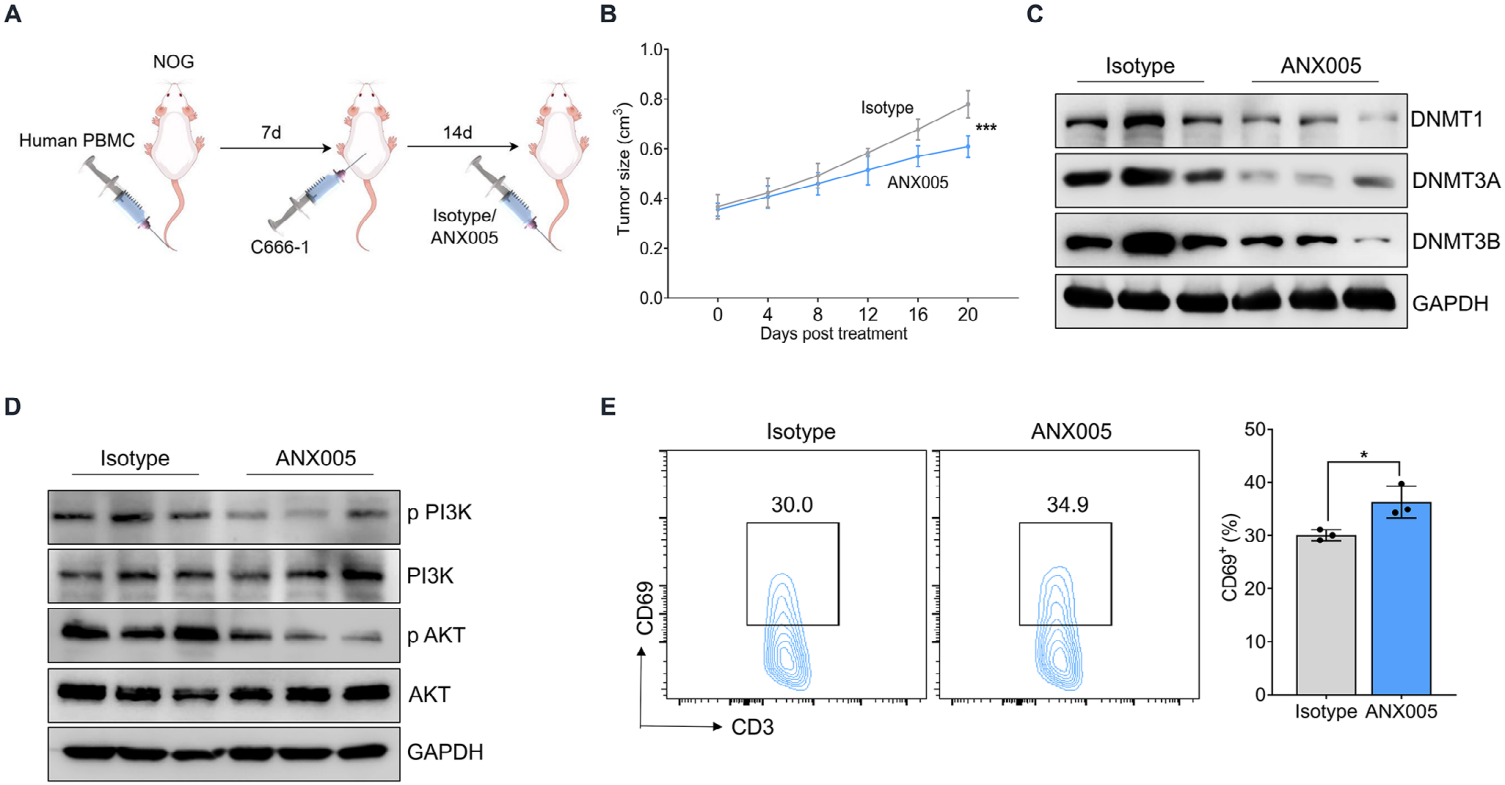
ANX005 treatment restricted NPC development in the humanized mouse model. A) NOG mice were tail vein injected with activated human PBMCs for 7 days. Then C666‐1 NPC cells were subcutaneously injected into humanized mice for another 7 days and treated with ANX005 or isotype every four days (n = 3). B) Tumor size was measured. C) DNMTs expression was evaluated by western blot. D) The activation of PI3K‐AKT signaling was measured by western blot assay. E) Flow cytometry analysis was performed to detect CD69 levels in T cells. The results were gated in CD3^+^ cells. ^*^ *p* < 0.05, ^***^ *p* < 0.01. Data from one representative experiment of three independent experiments are presented. Two‐tailed unpaired Student's *t*‐test was used to analyze the difference between the two groups. One‐way ANOVA was used to compare the differences among multiple groups.

Although our in vitro experiments have demonstrated the tumorigenic potential of C1q, its role in tumorigenesis under in vivo conditions remains to be elucidated. Therefore, we established an NPC humanized mouse model and treated the humanized mouse with ANX005, an anti‐C1q monoclonal antibody that has been involved in clinical trials (**Figure** **7**A). The results showed that ANX005 treatment restricted tumor development compared to the isotype counterparts (Figure 7B). Furthermore, tumor cells from ANX005‐treated mice exhibited impaired DNMTs expression (Figure 7C), indicating a decline in DNA methylation levels. Similarly, C1q blockade suppressed activation of PI3K‐AKT signaling, as indicated by western blot assay (Figure 7D). The immune cells from tumor lesions were also isolated, and we observed enhanced CD69 expression in T cells from ANX005‐treated mice, indicating improved T cell activation (Figure 7E). Taken together, these data demonstrated that ati‐C1q treatment restricted NPC development in vivo.

### 2.8 The Differentiation of C1q^+^ TAMs Possibly Acquired both M1 and M2 Polarization Condition

**Figure 8:**
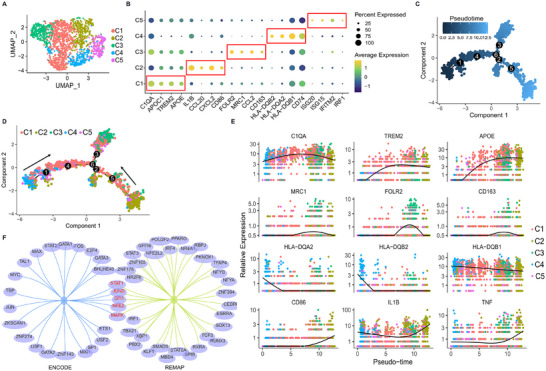
The differentiation of C1q^+^ TAMs possibly acquires both M1 and M2 polarization conditions. A) UMAP displayed the subclusters in the TAMs. B) Mark genes of each subcluster in the TAMs were shown. C) The Pseudotime analysis was performed. D) The Pseudotime analysis was displayed by the subclusters. E) The expression pattern of indicated genes in pseudo‐time was shown. F) A Venn diagram exhibited the common predicated transcription factor that regulated C1q^+^ TAMs polarization between ENCODE and REMAP datasets.

Although C1q^+^ TAMs have been characterized in various cancers, the differentiation of C1q^+^ TAMs has not been explored yet. To identify the possible differentiation trace of C1q^+^ TAMs, we clustered TAMs into five precise subtypes (**Figure** **8**A). Cluster 1 was identified as C1q^+^ TAMs, which expressed C1q, Apolipoprotein E (APOE), and Triggering Receptor Expressed on Myeloid cells 2 (TREM2). TAMs in cluster 2 expressed inflammatory cytokine IL‐1β and M1 macrophage marker CD86. Cluster 3 was marked with FOLR2 and highly expressed M2 macrophage markers MRC1 (CD206) and CD163, while expressing lower levels of C1q, APOE, and TREM2 than cluster 1. Cluster 4 was characterized by the expression of HLA‐II molecules, which are responsible for antigen presentation. Cluster 5 highly expressed interferon‐related genes, indicating a strong interferon response (Figure 8B). Pseudotime analysis was then performed to map the possible macrophage differentiation trace (Figure 8C). We assumed a double‐origin differentiation model in which C1q^+^ TAMs and FOLR2^+^ TAMs were derived from both antigen presentation TAMs and inflammatory TAMs (Figure 8D). The expression pattern of the marker genes in the differentiation trace was analyzed. Notably, C1q was the only marker gene of C1q^+^ TAMs and FOLR2^+^ TAMs that was expressed from both origins (Figure 8E), indicating that the expression of C1q wasn't the consequence of TAMs differentiation, while TAMs polarized into an immunosuppressive phenotype indeed boosted C1q production. Moreover, we explored the top 100 differential genes from C1q^+^ TAMs to the others and enriched the predominantly transcription factors (TFs) that regulated the expression of the differential genes. Surprisingly, despite the TFs that regulated the activation of macrophages (JUND), TFs of both M1 macrophage polarization (STAT1, SPI1) and M2 macrophage polarization (NFE2, MAFK) were enriched (Figure 8F). Taken together, these data indicated that the differentiation of C1q^+^ TAMs possibly acquired both M1 and M2 polarization conditions.

## 3 Discussion

The TME represents a distinctive niche characterized by continuous crosstalk among intertumoral cellular compartments. This dynamic cellular interplay within the TME plays a pivotal role in governing tumor progression and therapeutic response. Although the TME components of NPC have been extensively mapped, the nature of cellular communication remains unexplored. Our previous investigation discovered that NPC tumor cells play a pivotal role in driving the lipid‐mediated development and homeostasis of Regulatory T cells (Tregs).^[^ ^5^ ^]^ However, while our previous research shed light on these aspects, the communication and interactions between tumor cells and other stromal cells in the TME remained unexplored. Hence, the current study aimed to delve into the crosstalk between tumor cells and TAMs in NPC. Although dendritic cells (DCs) were observed to produce C1q as well in our sc‐RNA data, their contribution to the total C1q pool was minimal. In contrast, TAMs were identified as the predominant source of C1q. Consequently, our investigation focused on elucidating the role of TAMs‐derived C1q in tumorigenesis. The DC and macrophage conditional C1q knockout mice would help systematically dissect the distinct roles of these differentially sourced C1q populations in tumorigenic processes. Furthermore, C1q was not only produced in the TME but also enriched in the serum. Functionally, we consider that circulating C1q regulates tumorigenesis, as well as C1q derived from macrophages in the tumor tissue. Nevertheless, circulating C1q primarily engages in systemic immune functions, including complement activation and cellular debris clearance,^[^ ^17^ ^]^ leading to the limited amount of circulating C1q that might reach the tumor lesions compared with C1q expressed within the tumor. Besides, the tumor periphery, composed of dense stromal cells and extracellular matrix,^[^ ^18^ ^]^ coupled with the disorganized, dysfunctional tumor vasculature characterized by irregular morphology and impaired blood flow,^[^ ^19^ ^]^ significantly limits circulating infiltration into the TME. Therefore, we believe that although both circulating C1q and C1q derived from TAMs can regulate tumor development, circulating C1q is hard to permeate into tumor lesions.

We supposed that C1q^+^ TAMs modulate the malignancy, stemness, and epigenetic alterations of tumor cells based on our scRNA‐seq data and the TCGA bulk RNA‐seq data. Prior research has demonstrated that TAMs can enhance the malignant phenotype and stemness of tumor cells through the secretion of TGF‐β, C‐C motif chemokine ligand 2 (CCL‐2), and interleukin‐6 (IL‐6).^[^ ^20^ ^]^ In our study, we unveiled a novel mechanism whereby TAMs secreted C1q to drive tumorigenesis. Given that C1q is primarily produced by TAMs, our findings highlight a more targeted interaction between TAMs and tumor cells.

Subsequently, we identified DNA hypermethylation as a putative epigenetic regulatory mechanism through which C1q influenced tumor development. Aberrant DNA methylation is recognized as a crucial event in tumorigenesis, with DNA methylation inhibitor therapy not only impeding malignancy in tumor cells but also modulating the function of immune cells involved in adaptive immunity.^[^ ^21^ ^]^ Specifically, DNA methylation in the vicinity of the TSS hinders transcription, whereas methylation in the exon region reinforces transcription.^[^ ^22^ ^]^ Notably, genes belonging to the WNT and TGF‐β families exhibited methylation around the exon region in our data. WNTs and TGF‐β have been shown to induce an immunosuppressive M2 macrophage phenotype.^[^ ^16^, ^23^ ^]^ Consequently, we further demonstrated that DNA hypermethylated NPC cells induced by C1q reinforced the immunosuppressive phenotype of TAMs. Additionally, TGF‐β has been linked to the differentiation of immunosuppressive Tregs, Bregs, Cancer‐Associated Fibroblasts (CAFs), and N2 neutrophils.^[^ ^24^ ^]^ Similarly, WNTs promote the polarization of Tregs and CAFs.^[^ ^25^ ^]^ Collectively, we postulated a cellular network in which C1q secretion by TAMs triggers DNA hypermethylation in tumor cells, leading to the enhanced secretion of WNTs and TGF‐β, thereby shaping a more pronounced immunosuppressive TME. Our pan‐cancer analysis unveiled a positive correlation between C1q levels and the expression of various immune checkpoints across diverse cancers, underscoring the potent role of C1q in impeding anti‐tumor immune responses. We contemplated the potential of anti‐C1q therapy as an optimal adjunct target for immune checkpoint blockade therapy.

C1q has been identified to interact with various membrane receptors to initiate signaling pathways. Our study revealed that C1q binds to GPR17 to induce DNA hypermethylation of tumor cells. cC1QR, gC1QR and HMGB1 were the previously documented receptors of C1q.^[^ ^26^ ^]^ We discovered that both GPR17 and these receptors were expressed in the NPC RNA‐seq data. Interestingly, we only identified GPR17 coupling with C1q based on our immunoprecipitation protein spectrum data. We hypothesize that this observation may be attributed to differences in binding affinity between C1q and its various receptors. In our experimental procedure, C1q was incubated with NPC cells for only 4 h prior to membrane isolation and immunoprecipitation. It is possible that extending the incubation time could facilitate the identification of additional C1q‐interacting proteins. Furthermore, studies have demonstrated that blockade of C1qR on tumor cells inhibits tumor proliferation,^[^ ^27^ ^]^ suggesting that C1q may regulate tumorigenesis through multiple mechanisms. Therefore, targeting C1q directly appears to be a more effective therapeutic strategy compared to targeting the receptors of C1q.

Although we have demonstrated the effect of C1q on inducing tumor cell hypermethylation and inducing immunosuppressive TAMs, C1q has been found to regulate DCs maturation^[^ ^28^ ^]^ and antigen presentation^[^ ^28b^ ^]^ in the tumor microenvironment, proposing a dual role of C1q in the TME. To further prove the in vivo effect of C1q on NPC development, the in vivo mouse experiments were performed. Given the absence of both mouse NPC cell lines and primary NPC mouse models, the humanized NPC mouse model remains the most suitable NPC animal model. Our in vivo experiments demonstrated that pharmacological blockade of C1q using the neutralizing monoclonal antibody ANX005 significantly suppressed NPC progression. ANX005 has been used in a phase 3 trial of Guillain‐Barré syndrome^[^ ^29^ ^]^ and a phase 2 trial of Huntington's Disease (https://doi.org/10.1212/WNL.0000000000203217). Our results suggested that although C1q regulates both tumor cells and immune cells, the synthesized effect of C1q is pro‐tumor in NPC, which was consistent with the previous report that mice deficient in C1q displayed decreased tumor growth in clear‐cell renal cell carcinoma.^[^ ^30^ ^]^

The differentiation trajectory of C1q^+^ TAMs remains an area that warrants further exploration. TAMs are traditionally categorized into anti‐tumor M1‐like macrophages and pro‐tumor M2‐like macrophages based on the in vitro polarization model.^[^ ^31^ ^]^ However, macrophages are highly diverse and plastic. Recent single‐cell analyses of human and mouse samples have revealed that the status of macrophages is not a simple M1/M2 dichotomy, but a continuous spectrum. In some cases, M1 and M2 phenotypes of macrophages may express “opposing” markers at the same time in vivo. This discrepancy between in vitro and in vivo observations may stem from the inability of in vitro models to fully replicate the complexity of the in vivo tumor microenvironment, resulting in differences in biomarker expression patterns. Based on recent reports, TAMs have been subdivided into more precise subtypes in vivo, with C1q^+^TAMs/TREM2^+^TAMs being implicated in exerting immunosuppressive effects. In the context of NPC tissues, we identified two distinct subtypes of immunosuppressive TAMs: C1q^+^ TREM2^+^ TAMs and FOLR2^+^ CD206^+^ CD163^+^ TAMs, with the specific functions of these subtypes necessitating further investigation. Additionally, C1q^+^ TAMs were observed to co‐express established M1 and M2 markers,^[^ ^32^ ^]^ and transcription factor predictions highlighted the involvement of both M1‐ and M2‐related transcription factors in C1q^+^ TAMs differentiation. STAT1, a key transcription factor in M1 macrophage polarization, has been shown to induce C1q expression in macrophages.^[^ ^33^ ^]^ Our hypothesis suggests that C1q expression may be activated during the M1‐like TAM polarization phase, with subsequent training in the TME leading to the transition of M1‐like TAMs into M2‐like TAMs, characterized by the co‐expression of M1 and M2 markers, culminating in the differentiation of C1q^+^ TAMs. Further validation of this hypothesis across different tumor models is planned.

In conclusion, our study identifies C1q as a novel regulator mediating interactions between TAMs and tumor cells. C1q emerges as a potential therapeutic target for combination tumor therapy, offering promising avenues for future research and therapeutic interventions in cancer treatment.

## 4 Experimental Section

### Bioinformatics Assay

The 10×scRNA‐seq was performed as previously reported.^[^ ^3^ ^]^ 11 NPC patients and 3 NLH patients were enrolled in this study. The clinical information of each patient was presented in the “Table S1 (Supporting Information)”. A Seurat object was created by the Seurat R package (version 4.0). The cells containing under 25% mitochondrial counts or unique features between 200 and 5000 were analyzed. The keeping cells were clustered based on UMAP. The Pseudotime analysis was performed by the “Monocle2” R package. The transcription factor prediction was conducted by ChEA3 (https://maayanlab.cloud/chea3/). The public scRNA‐seq dataset TISCH (http://tisch.comp‐genomics.org/) was used for pan scRNA‐seq gene expression and survival analysis. Public bulk RNA‐seq data of NPC were obtained from GEO datasets (GSE61218, GSE12452, GSE102349) and OmicsDI datasets (EGAD00001009047). Public bulk RNA‐seq data of pan‐cancer were acquired from The Cancer Genome Atlas (TCGA) datasets. The survival data of NPC patients were provided by Professor Wei Dai at the University of Hong Kong. The GSEA analysis was performed for pathway enrichment in GO datasets. To enrich the pathway of C1q^+^ TAMs‐regulated tumor cells, differential genes between tumor cells from C1q^+^ TAMs high‐enriched patients and C1q^+^ TAMs low‐enriched patients in the scRNA‐seq data were analyzed and then subjected to pathway enrichment. C1q^+^ TAMs high and low were divided at the upper quartile and lower quartile. To enrich the common differential pathway between C1q high expression patients and C1q low expression patients in TCGA datasets, data of breast cancer, cholangiocarcinoma, and hepatocellular carcinoma were included. C1q high expression and low expression were divided at the upper quartile and the lower quartile. For DNA methylation‐seq, the DNA was extracted by Qiagen DNeasy Blood & Tissue Kit (69504, Qiagen, Germany). The experiments were performed by Infinium MethylationEPIC BeadChip. The 1217 human tumor suppressor genes were identified in TSGene database (https://bioinfo.uth.edu/TSGene/index.html).

### Animal Breeding and Treatments

The BALB/cAnN‐nu (Nude) mice were housed at a constant temperature (19–23 °C) and 55 ± 10% humidity and were kept under a 12 h light/dark cycle. All mice had free access to water and commercial feed. The animal experiments were approved by the Medical Ethics Committee of the University of Hong Kong Shenzhen Hospital. Six‐week‐old mice were used for experiments. All mice were euthanized with 5% isoflurane. This study exclusively examined male mice. It was unknown whether the findings were relevant for female mice. For the xenograft model, C666‐1 cells were treated with 1 µg mL^−1^ C1q (A099, CompTech, USA) or equal volume PBS as counterparts for three days in vitro, and then 1 × 10^6^ cells were subcutaneously injected into nude mice. The mice were euthanized three weeks post‐injection. For the lung metastasis model, 1 × 10^6^ GFP‐labeled cells were tail vein injected into nude mice. Mice were euthanized four weeks post‐injection, and GFP fluorescence was detected in lung tissues by animal live imaging to indicate tumor nodules. For the limited dilution assay, 1 × 10^5^ or 1 × 10^4^ treated cells were subcutaneously injected into nude mice, mice were euthanized three weeks post‐injection, and the tumor initiation frequency of CSCs was calculated by the limited dilution analysis in ELDA software (https://bioinf.wehi.edu.au/software/elda/index.html). In some cases, cells were cultured in the condition of 5 µM azacitidine (S1782, Selleck, China) or were transfected with AKT knockdown plasmids, and the mice were euthanized 4 weeks post‐injection. All mice were randomly assigned to each group.

### Establishment of NPC Humanized Mice Model

The humanized mouse model was established as previously reported.^[^ ^5^ ^]^ Briefly, PBMCs from healthy donors were isolated and activated by anti‐CD3/CD28 antibody (16‐0037‐81, 14‐0289‐82, Thermo Fisher Scientific, USA). 1 × 10^7^ of activated PBMCs were tail vein injected into each 5‐week‐old male NOG mouse. After seven days, 1 × 10^6^ of C666‐1 cells were subcutaneously injected into humanized mice for another seven days. 10 mg kg^−1^ ANX005 (Ab177901, Aladdin, China) or isotype (ab288147, Abcam, USA) was tail vein injected into humanized mice every four days. The tumor size was measured.

In some cases, C666‐1 cells were treated with 1µg mL^−1^ C1q for seven days. The cells were then harvested and subcutaneously injected into humanized mice for three weeks.

The tumor cells and immune cells from tumor tissues were isolated by percoll (45‐001‐748, Fisher Scientific, USA) density gradient centrifugation. Cells were resuspended in 40% peroll, then placed on top of 70% percoll. After centrifugated at 2500 rpm min^−1^ for 20 min at 22 °C, the buffy coat was identified as immune cells, and the cell pellet was identified as tumor cells.

### Cell Culture and Treatments

The cell lines involved in this investigation had been confirmed authentic by short tandem repeat (STR) profiling and tested for mycoplasma negativity by PCR analysis (C0301S, Beyotime Biotechnology, China). Cells were cultured in DMEM (11965092, Thermo Fisher Scientific, USA) supplemented with 10% fetal bovine serum (FBS) (10099141C, Thermo Fisher Scientific, USA), 100 U mL^−1^ penicillin, and 100 µg mL^−1^ streptomycin (15070063, Thermo Fisher Scientific, USA). Cells were maintained at 37 °C and 5% CO_2_. Plasmids were transfected through Lipo3000 (L3000150, Thermo Fisher Scientific, USA) based on the instructions. Poly‐clonal cells were involved in the experiments. In some cases, cells were treated with C1q protein purified from pooled normal human plasma (A099, CompTech, USA) or cultured in the condition of 5 µM azacitidine (S1782, Selleck, China). NP460 and C666‐1 cells were kindly provided by Professor Sai Wah Tsao (The University of Hong Kong, Hong Kong, China). All the sequences of primers involved in Crispr Cas9 knockout were uploaded as a supplemental file.

### CCK‐8 Assay

Cell proliferation and viability were detected by Cell Counting Kit‐8 (CCK‐8, Dojindo Molecular Technologies, Japan) based on the instructions. Briefly, cells were seeded into 96‐well plates. 10µl of CCK8 solution was added to the culture medium, followed by incubation at 37 °C for 2 h. The 450 nm absorbance was detected.

### Colony Formation Assay

After being cultured for 7 days, the cells were fixed with 4% paraformaldehyde and then stained with crystal violet at room temperature for 10 min. Colony numbers were evaluated.

### Migration Assay

Cells seeded into the upper chambers were cultured in a serum‐free medium. Medium containing 10% FBS was added to the lower chambers. The chambers were incubated at 37 °C for 24 h. The cells in the upper chamber were removed. The migrated cells were fixed with 4% paraformaldehyde and stained with crystal violet. The numbers of migrated cells were calculated.

### Wound Healing Assay

The cells were seeded and scratched with 1 mL pipette tips, then cultured in a serum‐free condition for 24 h. The migration rate was evaluated.

### RT‐qPCR Analysis

Total RNA was extracted with 1 mL TRIzol (15596018CN, Thermo Fisher Scientific, USA) based on the manufacturer's instructions. The cDNA was synthesized using a commercial kit (E047, novoprotein, China). Briefly, cDNA was synthesized at 42 °C for 5 min, 50 °C for 15 min, and 75 °C for 5 min. SYBR Green (208054, Qiagen, Germany) was applied for qPCR according to the following conditions: initial denaturation at 95 °C for 2 min, followed by 35 cycles of denaturation at 95 °C for 10 s and extension at 60 °C for 20 s. The expression of the target genes was normalized to GAPDH and calculated by the 2^−∆∆Ct^ method. All the sequences of primers involved in this study are provided in Table S2 (Supporting Information).

### Flow Cytometry

For macrophage‐tumor cell coculture experiments, THP‐1 cells were treated with 100ng mL^−1^ PMA for 24 h to polarize into macrophages. Tumor cells were treated with 1µg mL^−1^ C1q for 48 h in a 0.4 µm pore insert and then replaced with a refreshed C1q‐free medium. The inserts were cocultured with PMA‐derived macrophages for 48 h.

For the T cell activation test, Jurkat cells were activated by 1 µg mL^−1^ anti‐CD3/CD28 antibody (16‐0037‐81/16‐0289‐81, Thermo Fisher Scientific) for 24 h, then cocultured with tumor cell cocultured macrophages for another 48 h. Activated Jurkat cells were incubated with 1 µg mL^−1^ C1q for 48 h to investigate the direct effect of C1q on T cell activation.

Indicated cells were stained with anti‐CD44 (17‐0441‐82, Thermo Fisher Scientific), CD69 (25‐0699‐42, Thermo Fisher Scientific), CD163 (12‐1639‐42, Thermo Fisher Scientific), CD206 (17‐2069‐42, Thermo Fisher Scientific), PD‐L1 (25‐5983‐42, Thermo Fisher Scientific) antibodies, at 4 °C in the dark for 30 min. A LIVE/DEAD Fixable Violet Dead Cell Stain Kit (L34966, Thermo Fisher Scientific, USA) was stained to identify live cells.

For Edu assay, the experiment was performed with an Edu staining kit (C0071, Beyotime Biotechnology, China). Briefly, the Edu solution was added to the culture medium and incubated at 37 °C for 2 h. Cells were then digested and fixed with 4% paraformaldehyde for 10 min. After permeabilizing with 0.5% Triton X‐100 for 10 min, the cells were incubated with the reaction buffer for 30 min at 37 °C.

Cell apoptosis was detected by an Annexin V/PI apoptosis kit (70‐AP101‐100, MultiScience, China). Cells were incubated with Annexin V/PI solution at room temperature for 10 min before detection.

Cellular calcium level was detected by a Fluo‐4 AM probe (S1060, Beyotime Biotechnology, China). Cells were incubated with 1 µM staining solution at 37 °C for 30 min.

All the flow cytometry results were detected by the flow cytometer and analyzed by the FlowJo.v10 software.

### Sphere Formation Assay

Cells were cultured in serum‐free DMEM/F12 medium (11330032, Thermo Fisher Scientific) contained 100 × N2 (17502001, Thermo Fisher Scientific), 50 × B27 (17504044, Thermo Fisher Scientific), 5 µg mL^−1^ insulin (11376497001, USA), 20 ng mL^−1^ epidermal growth factor (AF‐100‐15, Peprotech, USA), 10 nM fibroblast growth factor (100‐18B, Peprotech), and 0.4% BSA. The cells were cultured in low attachment 24‐well plates for 7 days.

### Immunoprecipitation and Western Blot

Whole‐cell lysates were extracted by cell lysis buffer (P0013, Beyotime Biotechnology) followed by quantification by the BCA assay (23225, Thermo Fisher Scientific). For the immunoprecipitation assay, membrane protein was extracted (P0033, Beyotime Biotechnology) and was incubated with 1 µg antibody at 4 °C overnight, then incubated with 20 µl protein A/G agarose (sc‐2003, Santa Cruz Biotechnology, USA) for 2 h at 4 °C. The eluted precipitants were determined by SDS‐PAGE.

For western blot analysis, SDS‐PAGE was performed, and the samples were then transferred onto PVDF membranes. The membranes were blocked by 5% BSA for 1 h at room temperature, followed by incubating with the indicated primary antibodies at 4 °C overnight. The next day, the membranes were incubated with HRP‐conjugated secondary antibody at room temperature for 1 h. For GPR17 detection, the membrane protein was extracted. Antibodies involved in this investigation were as follows: C1q (sc‐53544, Santa Cruz Biotechnology), DNMT1 (24206‐1‐AP, Proteintech, USA), DNMT3A (20954‐1‐AP, Proteintech), DNMT3B (26971‐1‐AP, Proteintech), PI3K (67071‐1‐Ig, Proteintech), AKT (10176‐2‐AP, Proteintech), phospho‐AKT (66444‐1‐Ig, Proteintech), GPR17 (13416‐1‐AP, Proteintech), GAPDH (10494‐1‐AP, Proteintech), phospho‐PI3K (AF3242, Affinity, USA), ATP1A1 (sc‐71639, Santa Cruz Biotechnology), HRP‐conjugated Goat Anti‐Mouse IgG(H^+^L) (SA00001‐1, Proteintech), HRP‐conjugated Goat Anti‐Rabbit IgG(H^+^L) (SA00001‐2, Proteintech).

### Immunofluorescence

Cells were fixed with 4% paraformaldehyde for 10 min. After blocking with goat serum for 1 h, the cells were stained with the indicated primary antibodies overnight at 4 °C. The cells were then incubated with goat anti‐mouse IgG (H^+^L) (Alexa Fluor 488) (A‐11001, Thermo Fisher Scientific) and goat anti‐rabbit IgG (H^+^L) (Alexa Fluor 555) (A‐21428, Thermo Fisher Scientific) antibodies for 1 h at 37 °C. The nucleus was stained with DAPI for 15 min at 37 °C.

For Dil staining, 5 µM staining solution (C1036, Beyotime Biotechnology) was added to the culture medium at 37 °C for 30 min. The cells were then fixed and blocked for primary antibody incubation.

The images were obtained with a 63× oil immersion objective on a LSM900 Carl Zeiss Microscope. The colocalization was quantized by the ZEN system.

### Statistical Analysis

GraphPad Prism 10.1.2 was used for statistical analyses. The data are presented as the mean ± standard deviation (SD). Two‐tailed unpaired Student's *t*‐test was used to analyze the difference between the two groups. One‐way ANOVA was used to compare the differences among multiple groups. Kaplan–Meier and log‐rank tests were used to determine survival rates. The correlations between gene expression were assessed using Pearson's test. *p*< 0.05 was considered to indicate a statistically significant difference. Sample sizes were determined as follows: for in vitro experiments, n = 3 biological replicates were used; for in vivo experiments, the specific sample size (n) for each experimental group is provided in the figure legend. All experiments were independently repeated in triplicate.

## Conflict of Interest

The authors declare no conflict of interest.

## Author Contributions

Y.L. and C.H. contributed equally to this work. Y.L. and X.G. designed experiments. Y.L., M.L., W.L., and L.B. performed the experiments. Y.L., C.H., and S.Z. analyzed the data. Y.T., S.L., B.Z., L.G., and H.W. provided technical support. Y.L. and X.G. wrote the paper. X.G., Y.L., Y.T., S.L., and H.W. provided the grant support. All authors reviewed and approved the manuscript prior to submission.

## Supporting information



Supporting Information

## Data Availability

The data that support the findings of this study are available from the corresponding author upon reasonable request.
