# Supplementary material for: C1q+ Macrophage–Tumor Cell Interaction Promoted Tumorigenesis via GPR17/PI3K/AKT Pathway Induced DNA Hypermethylation in Nasopharyngeal Carcinoma
Source: Adv Sci (Weinh). 2025 Apr 2;12(26):2503434. doi: 10.1002/advs.202503434 (PMC12244503; doi:10.1002/advs.202503434)
Supplement: Supplementary file 1 — Supporting Information [file ADVS-12-2503434-s001.docx]

**Supplemental information**

**C1q^+^ Macrophage - Tumor Cell Interaction Promoted Tumorigenesis via GPR17/PI3K/AKT Pathway Induced DNA Hypermethylation in Nasopharyngeal Carcinoma**

Yunzhi Liu^1,2,#^, Cuicui Huang^1,3, #^, Min Luo^1^, Wenfu Lu^4^, Baifeng Zhang^1-3^, Lu Bai^1,3^, Shuyue Zheng^1,3^, Yanan Tan^1-3^, Shanshan Li^1-3^, Huali Wang^1-3^, Lanqi Gong^1, 2^, and Xinyuan Guan^1-3, 5^*

^#^ Yunzhi Liu and Cuicui Huang contributed equally to this manuscript

***Correspondence**:

Xinyuan Guan, Department of Clinical Oncology, Li Ka Shing Faculty of Medicine, The University of Hong Kong, Hong Kong, China.

Email: [xyguan@hku.hk](mailto:xyguan@hku.hk)

**
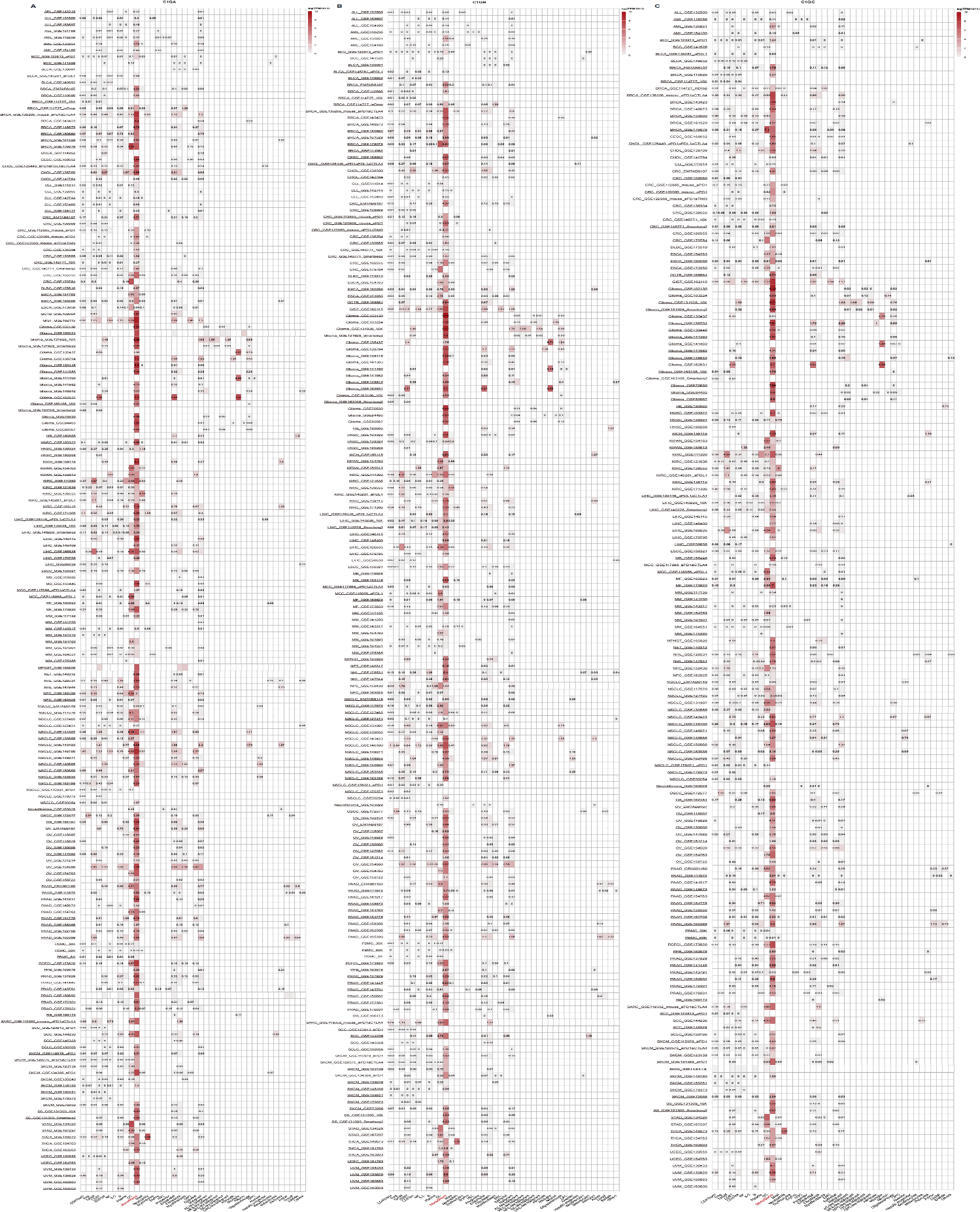
Supplemental Figure S1**

**Fig. S1 C1q was predominantly expressed in the macrophages.** C1QA (A), C1QB (B) and C1QC (C) expression pattern in pan scRNA-seq data was analyzed based on the TISCH database.

**Supplemental Figure S2**

**
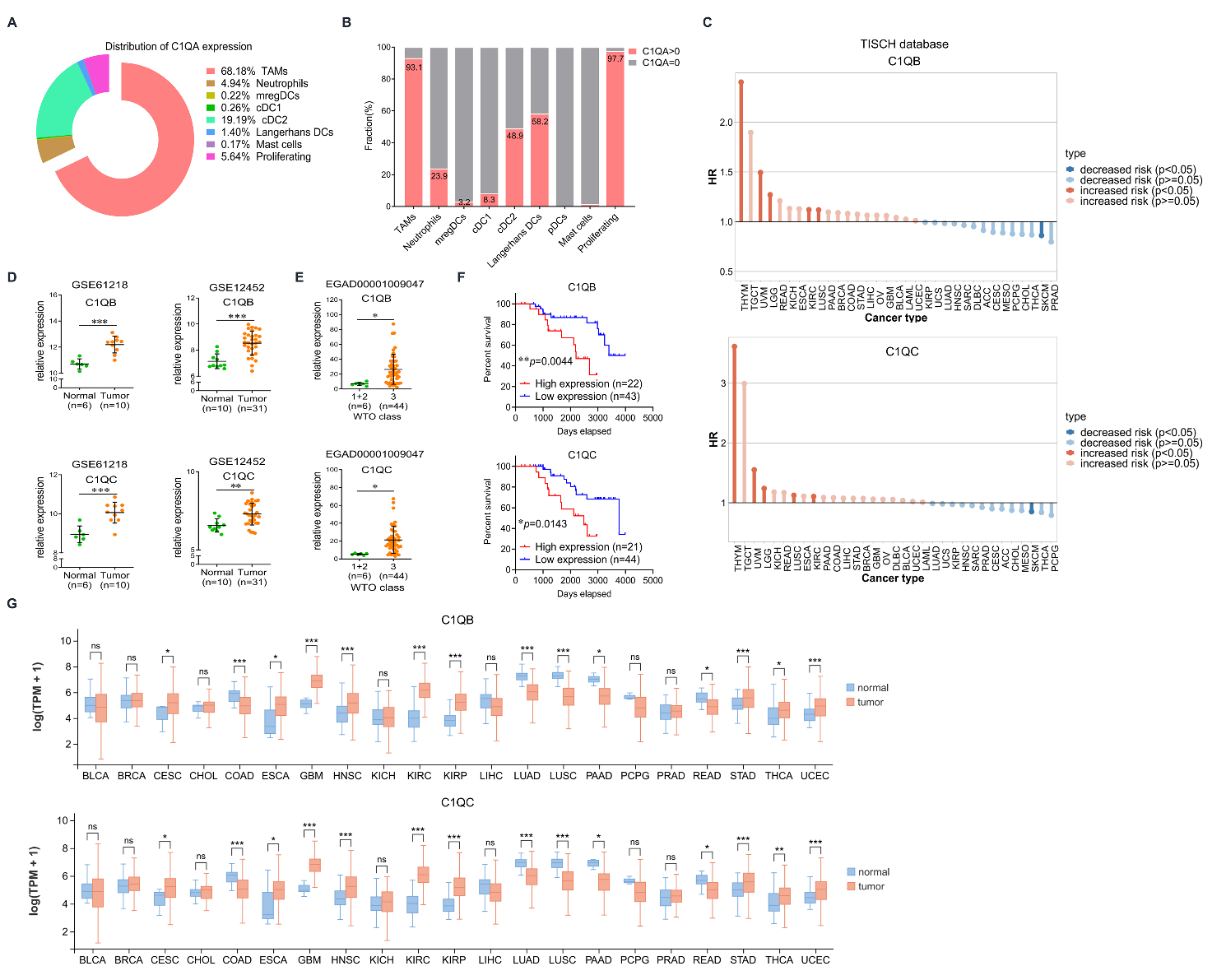
**

**Fig. S2 C1q expression was associated with NPC development.** (A)The distribution of C1QA expression was calculated. (B) The ratio of C1QA positive cells in each cell clusters were indicated. (C) Survival analysis of pan-cancer scRNA-seq data with high C1QB/C1QC expression and low C1QB/C1QC expression based on the TISCH database. (D) Expression levels of C1QB and C1QC in tissues from NPC patients and non-tumor individuals in the GSE61218 and GSE12452 datasets. (E) Expression levels of C1QB and C1QC in tissues from NPC tissues classified as stage Ⅲ and tissues from patients classified as stage Ⅰ and Ⅱ in the EGAD00001009047 dataset. (F) Survival analysis of NPC patients with high C1QB/C1QC expression and low C1QB/C1QC expression. (G) Expression levels of C1QB and C1QC in pan-cancer analysis based on TCGA database. NS: not significant, **p*<0.05, ***p*<0.01, ****p*<0.001. Kaplan–Meier and log-rank tests were used to determine survival rates.

**Supplemental Figure S3**

**
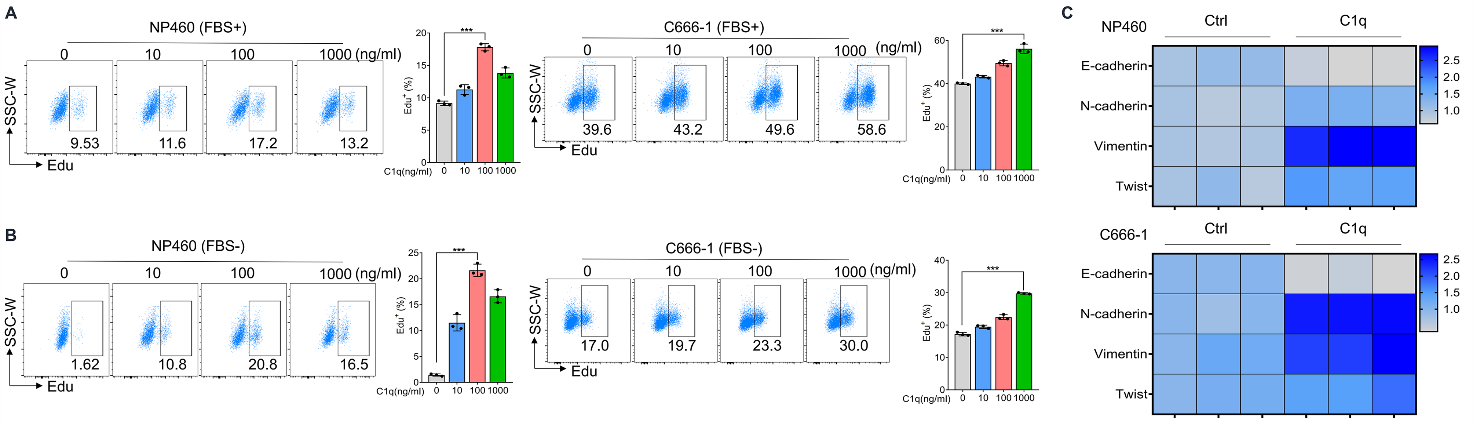
**

**Fig. S3 C1q enhanced the malignant phenotype of NPC cells.** (A-B) NP460 and C666-1 cells were treated with indicated concentrations of C1q for 24 hours. Edu assay was employed to detect cell proliferation. (C) NP460 and C666-1 cells were treated with 1 μg/ml C1q for 24 hours. RT-qPCR analysis was conducted to detect the mRNA levels of EMT-related genes. ****p*<0.001. Data from one representative experiment of three independent experiments are presented. Two tailed unpaired Student’s t test was used to analyze the difference between the two groups.

**Supplemental Figure S4**


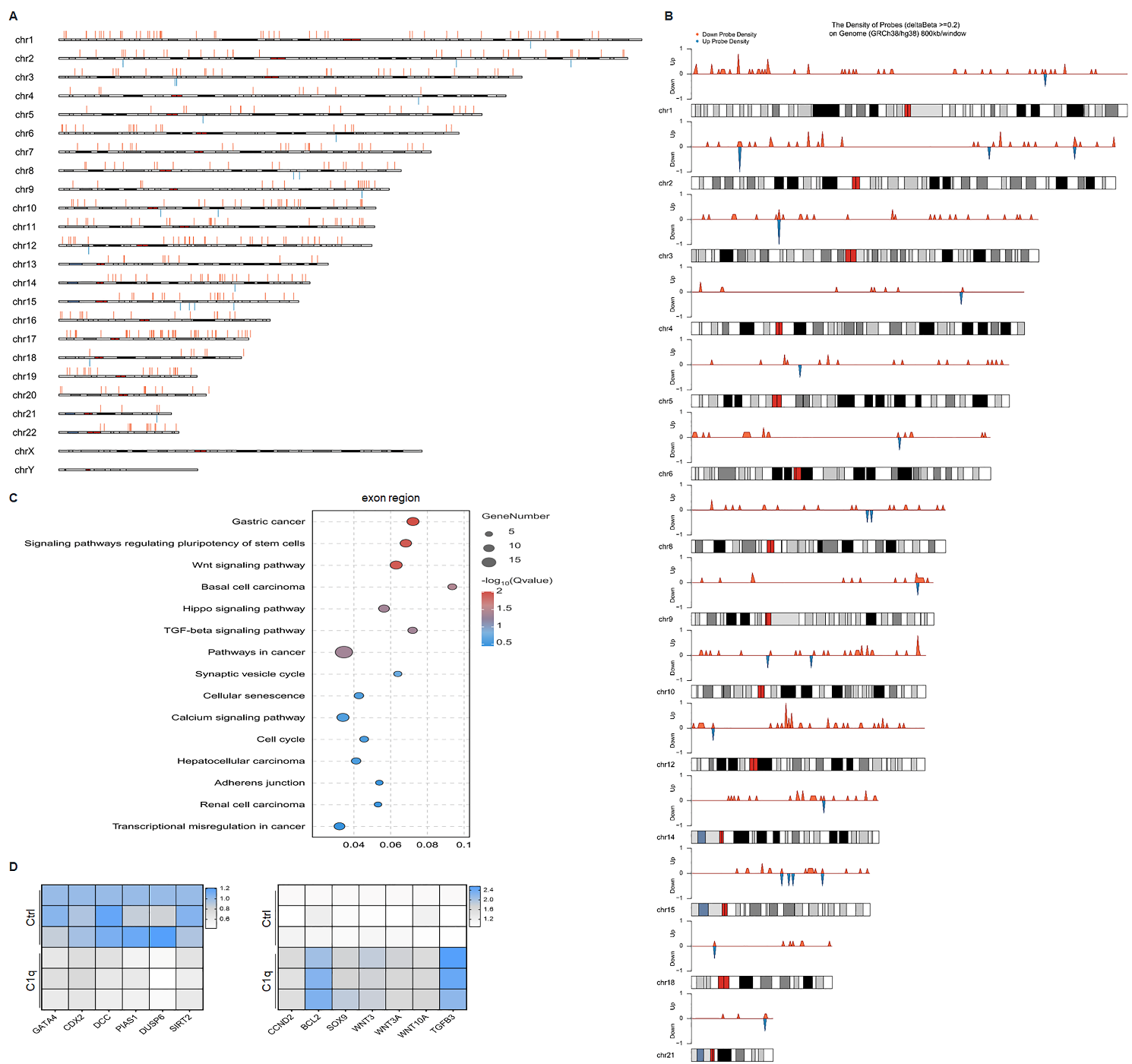


**Fig. S4 C1q elicited DNA hypermethylation of NPC cells.** (A-C) C666-1 cells were treated with 1μg/ml C1q for 24 hours to conduct DNA methylation-seq. (A) Chromosome distribution of the differential DNA methylation site was displayed. (B) The density of the probe on each chromosome was shown. (C) The pathway enrichment of differential genes that were methylated at the exon region. (D) C666-1 cells were treated with 1μg/ml C1q for 24 hours, RT-qPCR analysis was conducted to detect the mRNA levels of indicated genes.

**Supplemental Figure S5**

**
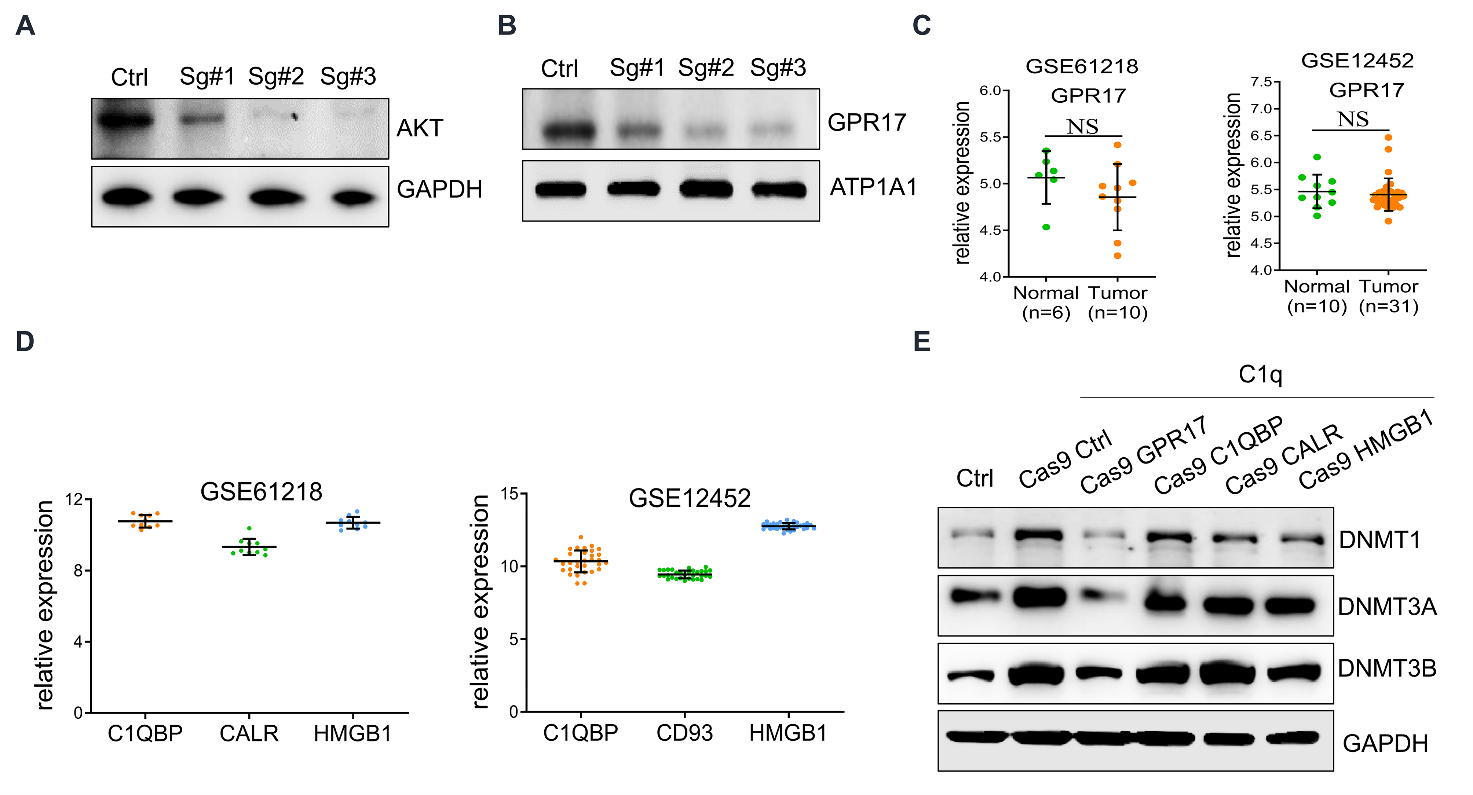
**

**Fig. S5 GPR17 was responsible for C1q induced DNA hypermethylation in NPC cells.** C666-1 cells were transfected with CRISPR/Cas9 AKT (A) or GPR17 (B) knockdown plasmids. A western blot assay was performed to evaluate protein expression, and the most efficient sgDNA was picked for further experiments. For GPR17 detcetion, membrane protein was extracted. (C) Expression levels of GPR17 in tissues from NPC patients and non-tumor individuals in the GSE61218 and GSE12452 datasets. (D) Expression levels of C1QBP, CALR and HMGB1 in tissues from NPC patients in the GSE61218 and GSE12452 datasets. (E) Western blot assay was performed to detect the expression of DNMTs. NS: not significant. Data from one representative experiment of three independent experiments are presented.

**Supplemental Figure S6**


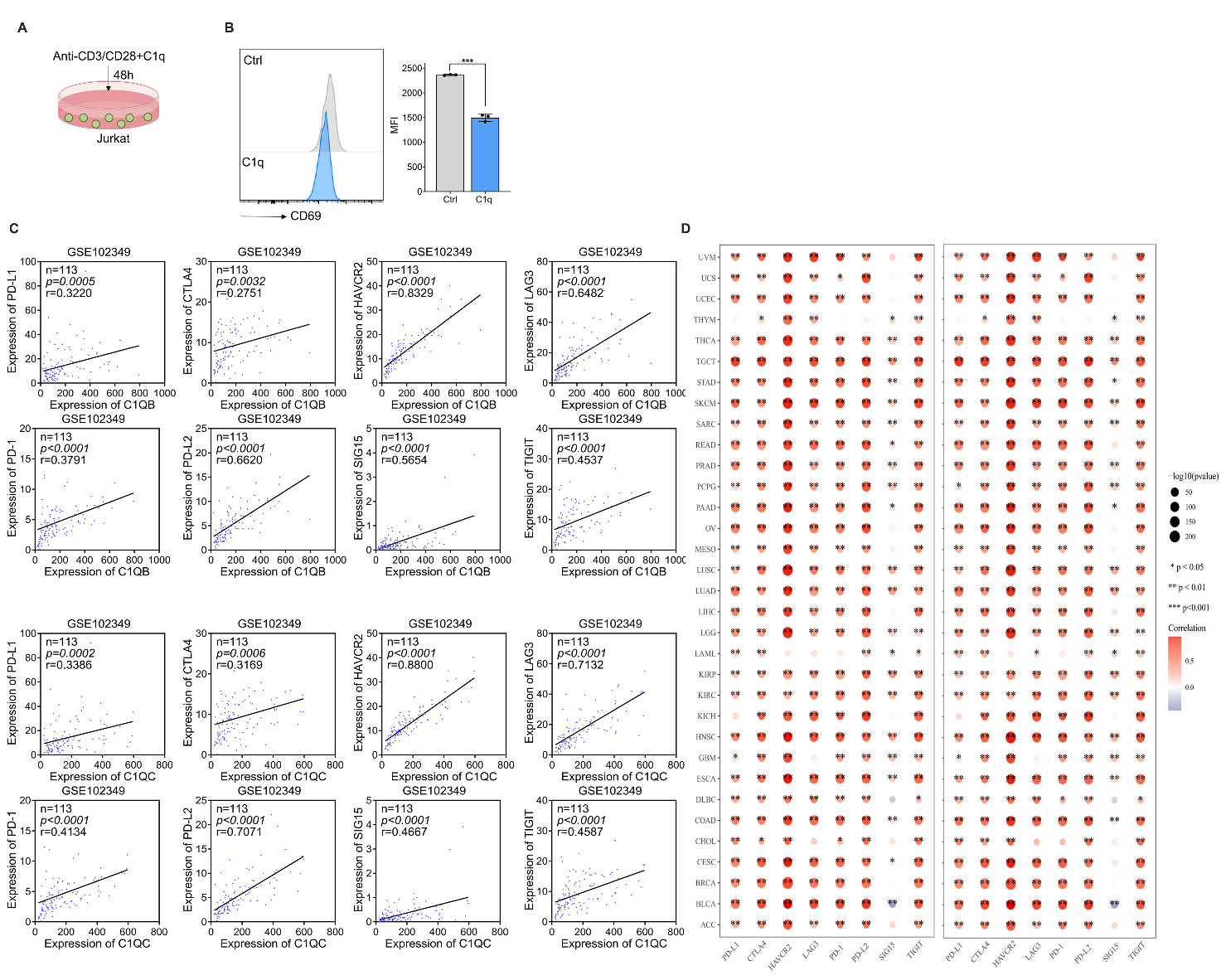


**Fig. S6 C1q level was positively correlated to the expression of immune checkpoints.** (A) Activated Jurkat cells were incubated with 1μg/ml C1q for 48 hours. (B) The CD69 level was detected by the flow cytometry assay. (C) The correlation between C1QB/C1QC expression and immune checkpoint expression in NPC patients was analyzed based on the GSE102349 dataset. (D) The correlation between C1QB/C1QC level and immune checkpoint expression in pan-cancer was analyzed based on the TCGA database. ****p*<0.01. Data from one representative experiment of three independent experiments are presented. Two tailed unpaired Student’s t test was used to analyze the difference between the two groups.

**Supplementary Table**

**Tab.S1 The clinical information of patients enrolled in the scRNA-seq.**

| Patient ID | Tissue type | Stage | EBV Status |
| --- | --- | --- | --- |
| 1 | NPC | Ⅲ | Negative |
| 2 | NPC | Ⅲ | Positive |
| 3 | NPC | Ⅲ | Positive |
| 4 | NPC | Ⅳ | Positive |
| 5 | NPC | Ⅰ | Negative |
| 6 | NPC | ⅣB | Positive |
| 7 | NPC | Ⅲ | Negative |
| 8 | NPC | ⅣA | N/A |
| 9 | NPC | Ⅲ | Positive |
| 10 | NPC | Ⅲ | Positive |
| 11 | NPC | Ⅲ | Negative |
| 12 | NLH | N/A | N/A |
| 13 | NLH | N/A | N/A |
| 14 | NLH | N/A | N/A |

**Tab.S2 Primers for the target genes.**

| Gene | Forward primer (5’🡪3’) | Reverse primer (5’🡪3’) |
| --- | --- | --- |
| (h) E-cadherin | CGAGAGCTACACGTTCACGG | GGGTGTCGAGGGAAAAATAGG |
| (h) N-cadherin | TCAGGCGTCTGTAGAGGCTT | ATGCACATCCTTCGATAAGACTG |
| (h) Vimentin | GACGCCATCAACACCGAGTT | CTTTGTCGTTGGTTAGCTGGT |
| (h) Twist | GTCCGCAGTCTTACGAGGAG | GCTTGAGGGTCTGAATCTTGCT |
| (h) CD44 | CTGCCGCTTTGCAGGTGTA | CATTGTGGGCAAGGTGCTATT |
| (h) Bmi1 | CCACCTGATGTGTGTGCTTTG | TTCAGTAGTGGTCTGGTCTTGT |
| (h) Oct3/4 | CTTGAATCCCGAATGGAAAGGG | GTGTATATCCCAGGGTGATCCTC |
| (h) Sox2 | GCCGAGTGGAAACTTTTGTCG | GGCAGCGTGTACTTATCCTTCT |
| (h) Nanog | TTTGTGGGCCTGAAGAAAACT | AGGGCTGTCCTGAATAAGCAG |
| (h) DNMT1 | AGGCGGCTCAAAGATTTGGAA | GCAGAAATTCGTGCAAGAGATTC |
| (h) DNMT3A | CCGATGCTGGGGACAAGAAT | CCCGTCATCCACCAAGACAC |
| (h) DNMT3B | AGGGAAGACTCGATCCTCGTC | GTGTGTAGCTTAGCAGACTGG |
| (h) GATA4 | CGACACCCCAATCTCGATATG | GTTGCACAGATAGTGACCCGT |
| (h) CDX2 | GACGTGAGCATGTACCCTAGC | GCGTAGCCATTCCAGTCCT |
| (h) DCC | ACCCAAGCTGGCTTTTGTACT | TGTGACGGCATCAGAAGGTTC |
| (h) PIAS1 | ACAGTGCGGAACTAAAGCAAA | GGACTTGAATGTACGTTGGGG |
| (h) DUSP6 | GAAATGGCGATCAGCAAGACG | CGACGACTCGTATAGCTCCTG |
| (h) SIRT2 | TGCGGAACTTATTCTCCCAGA | GAGAGCGAAAGTCGGGGAT |
| (h) CCND2 | ACCTTCCGCAGTGCTCCTA | CCCAGCCAAGAAACGGTCC |
| (h) BCL2 | GGTGGGGTCATGTGTGTGG | CGGTTCAGGTACTCAGTCATCC |
| (h) SOX9 | AGCGAACGCACATCAAGAC | CTGTAGGCGATCTGTTGGGG |
| (h) WNT3 | CTCGCTGGCTACCCAATTTG | AGGCTGTCATCTATGGTGGTG |
| (h) WNT3A | AGCTACCCGATCTGGTGGTC | CAAACTCGATGTCCTCGCTAC |
| (h) WNT10A | GGTCAGCACCCAATGACATTC | TGGATGGCGATCTGGATGC |
| (h) TGFB3 | ACTTGCACCACCTTGGACTTC | GGTCATCACCGTTGGCTCA |
| (h) GAPDH | GGAGCGAGATCCCTCCAAAAT | GGCTGTTGTCATACTTCTCATGG |
